# Supplementary figures and images for: The USP12/46 deubiquitinases protect integrins from ESCRT-mediated lysosomal degradation
Source: EMBO Rep. 2024 Nov 6;25(12):5687–718. doi: 10.1038/s44319-024-00300-9 (PMC11624278; doi:10.1038/s44319-024-00300-9)

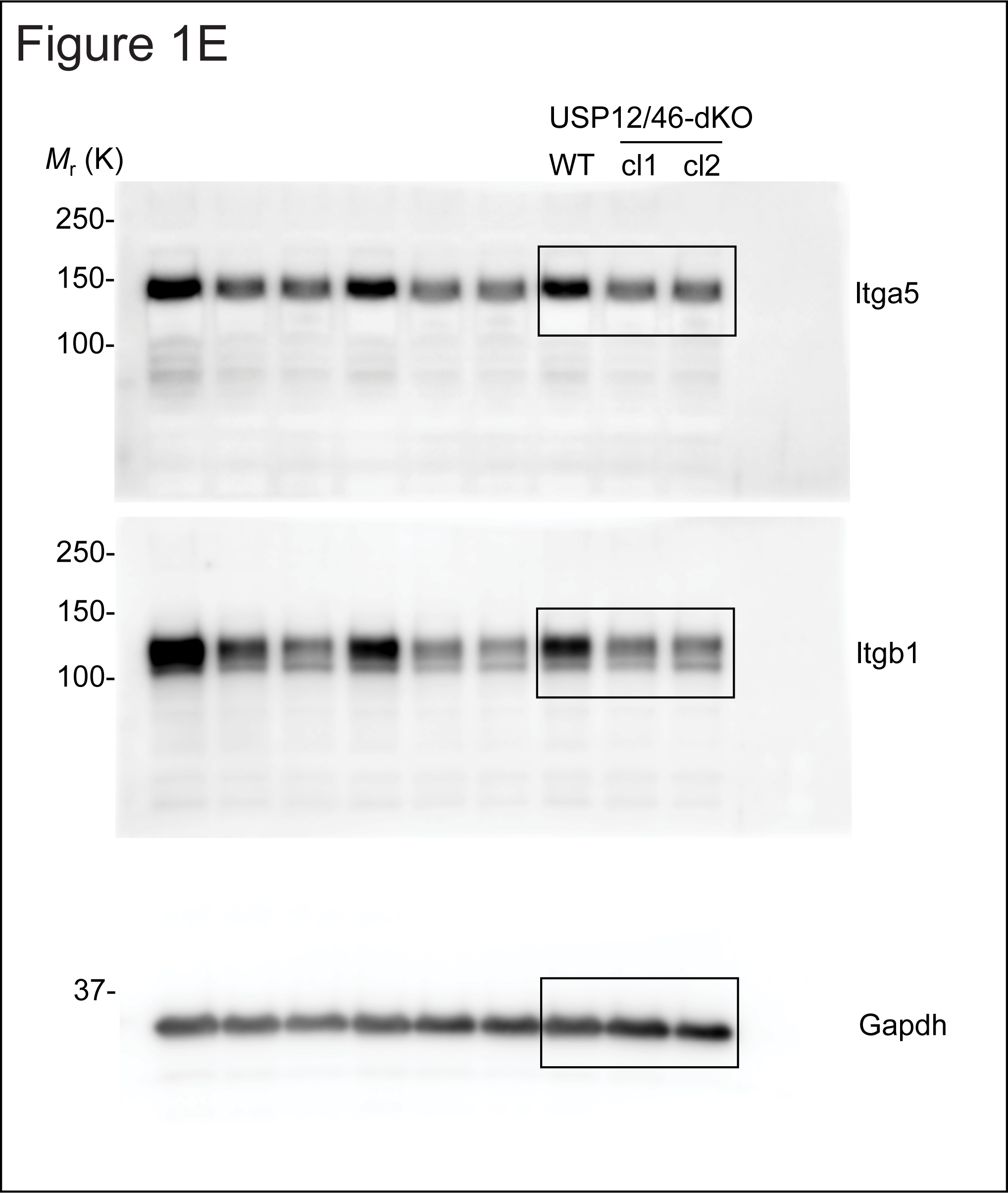

Supplement: Supplementary file 4 — Source data Fig. 1 [file 44319_2024_300_MOESM4_ESM.zip › Figure 1/1E/Figure 1E.tif]

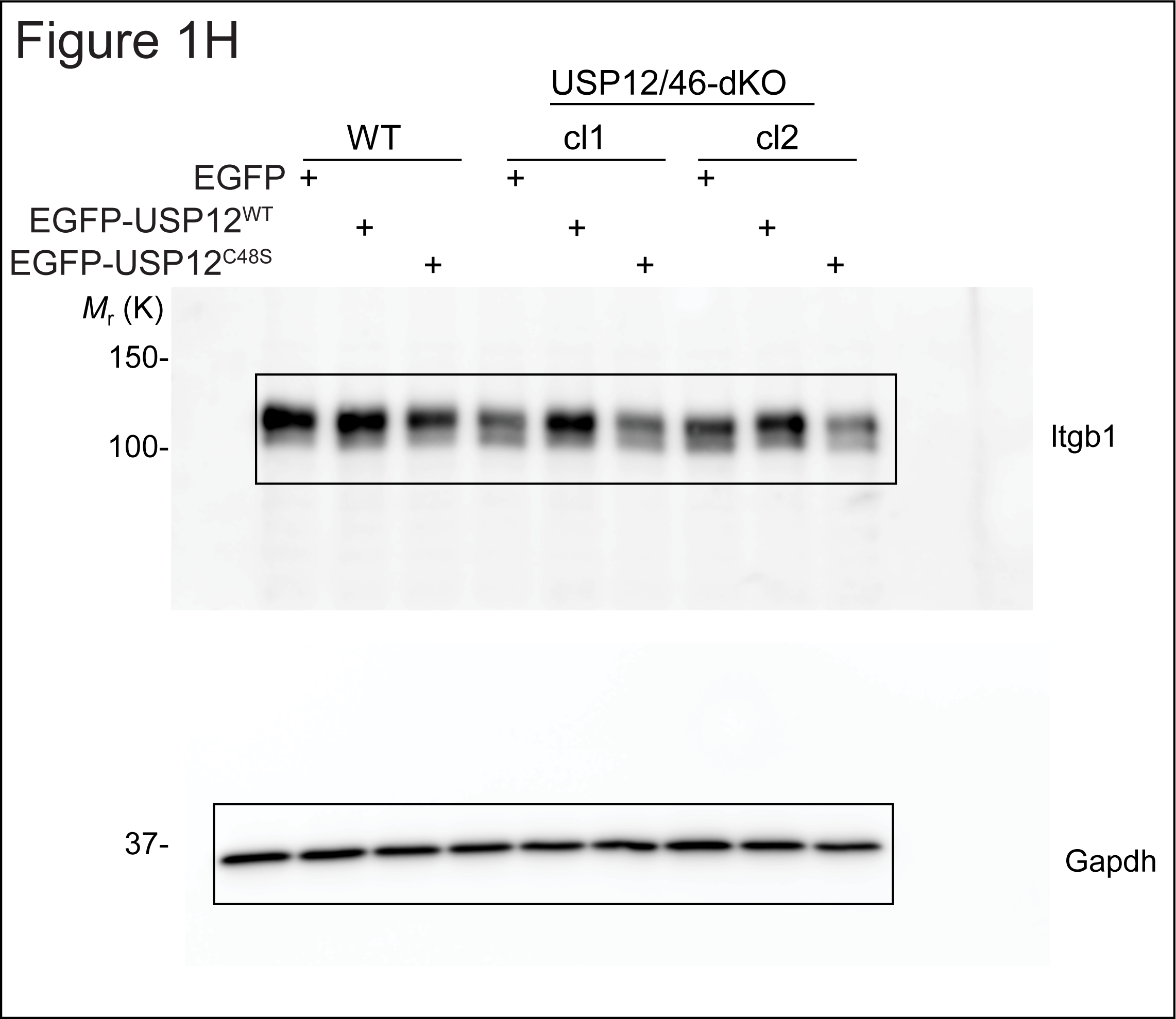

Supplement: Supplementary file 4 — Source data Fig. 1 [file 44319_2024_300_MOESM4_ESM.zip › Figure 1/1H/Figure 1H.tif]

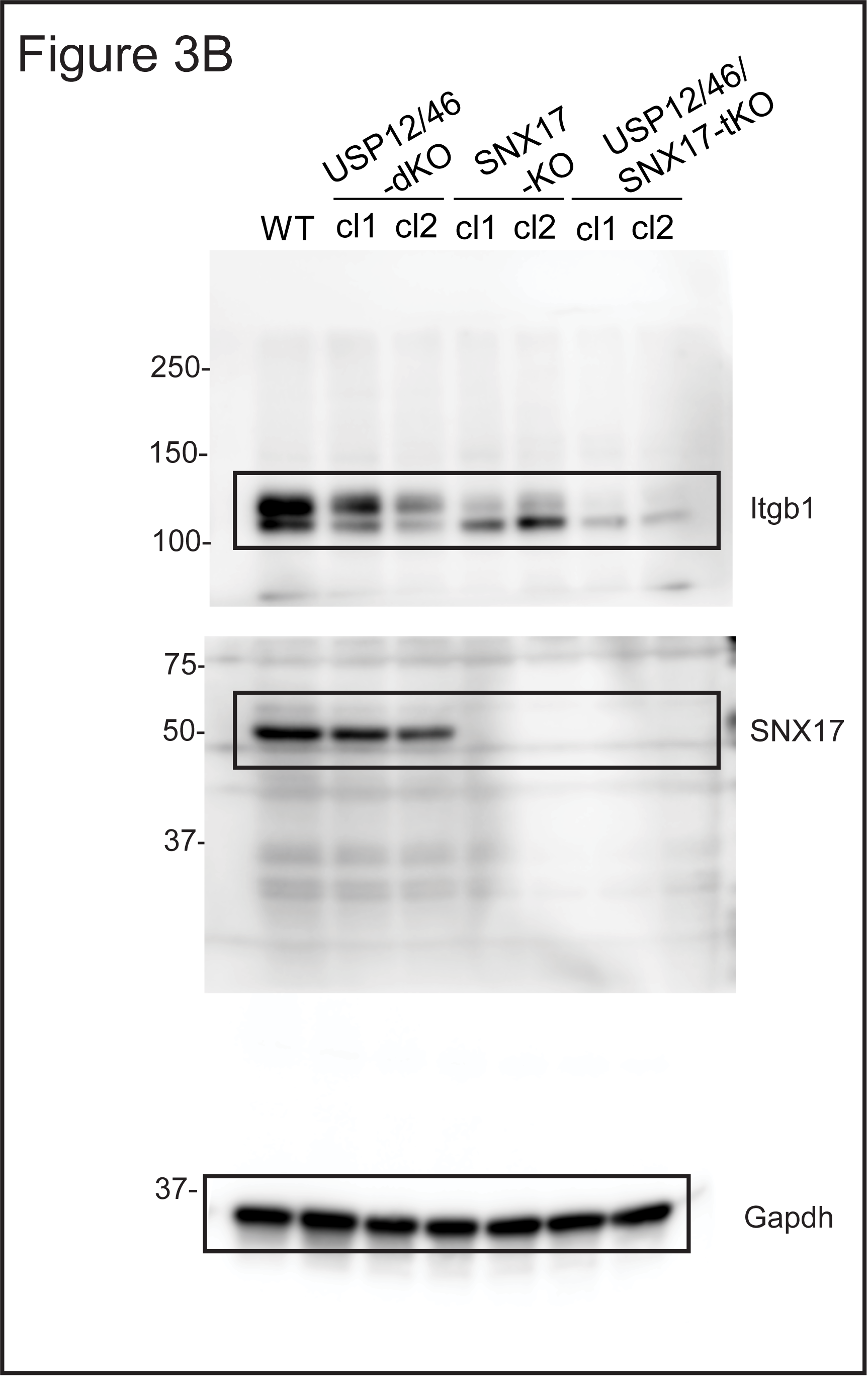

Supplement: Supplementary file 6 — Source data Fig. 3 [file 44319_2024_300_MOESM6_ESM.zip › Figure 3/3B/Figure 3B.tif]

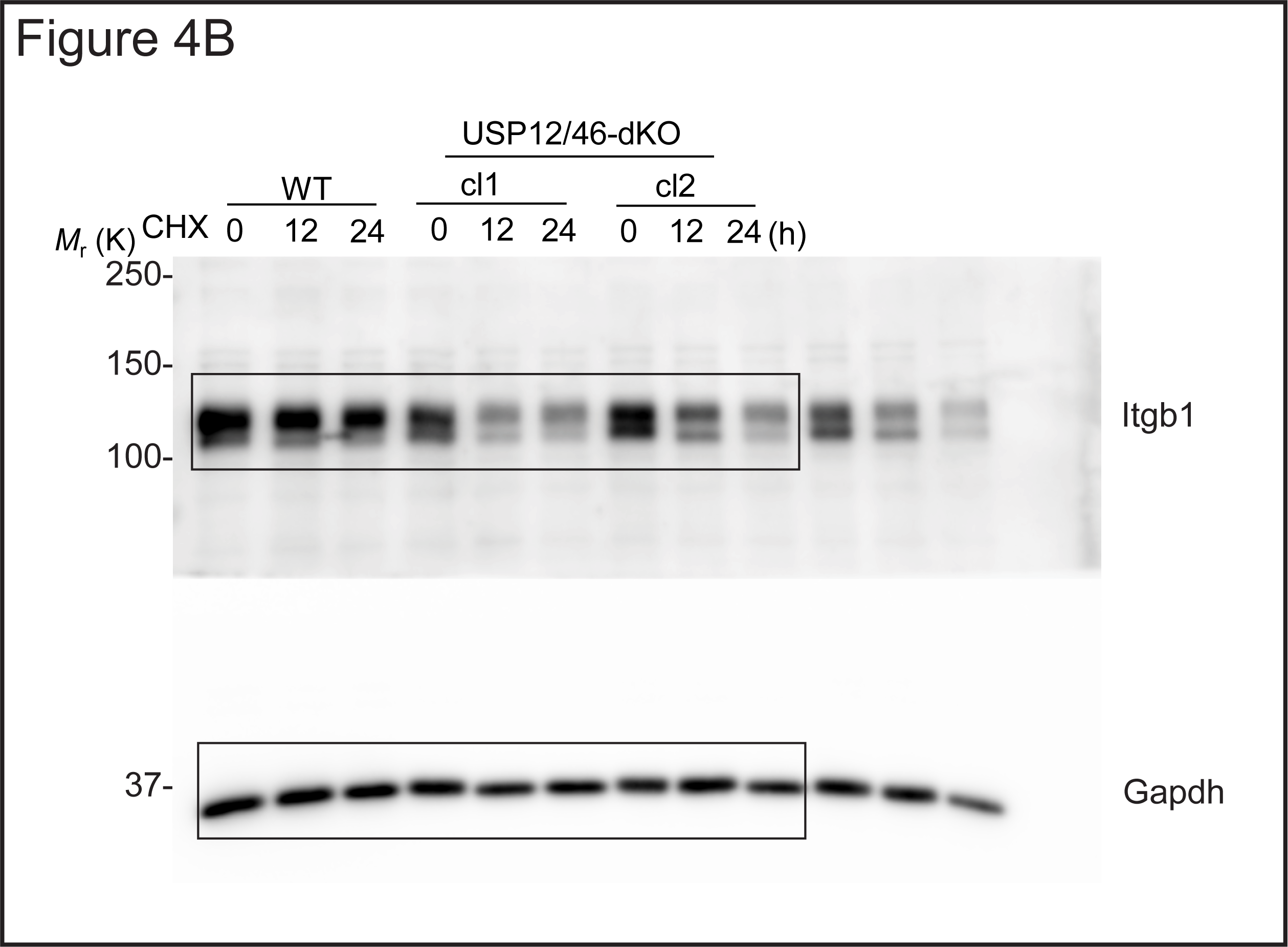

Supplement: Supplementary file 7 — Source data Fig. 4 [file 44319_2024_300_MOESM7_ESM.zip › Figure 4/4B/Figure 4B.tif]

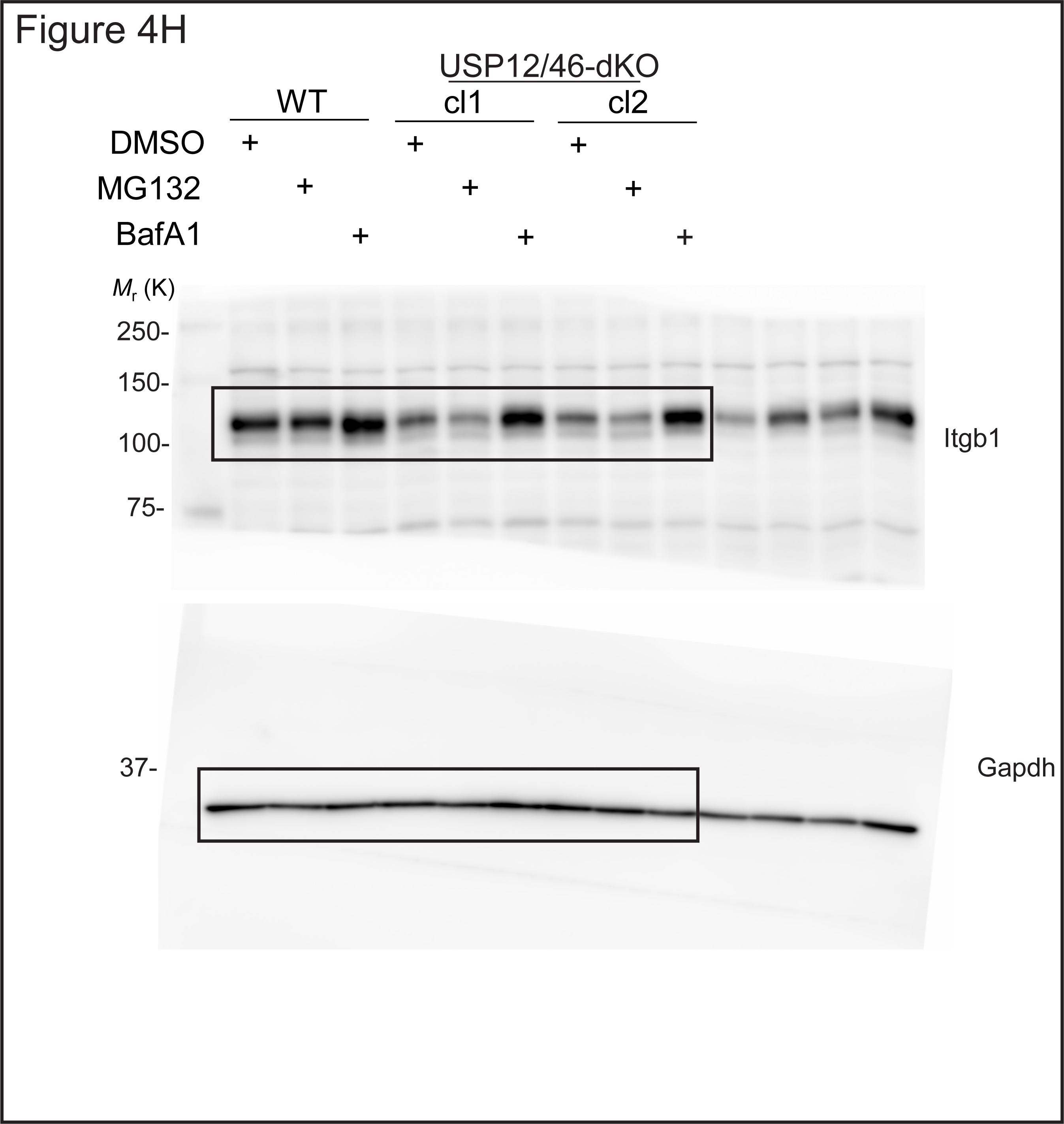

Supplement: Supplementary file 7 — Source data Fig. 4 [file 44319_2024_300_MOESM7_ESM.zip › Figure 4/4H/Figure 4H.tif]

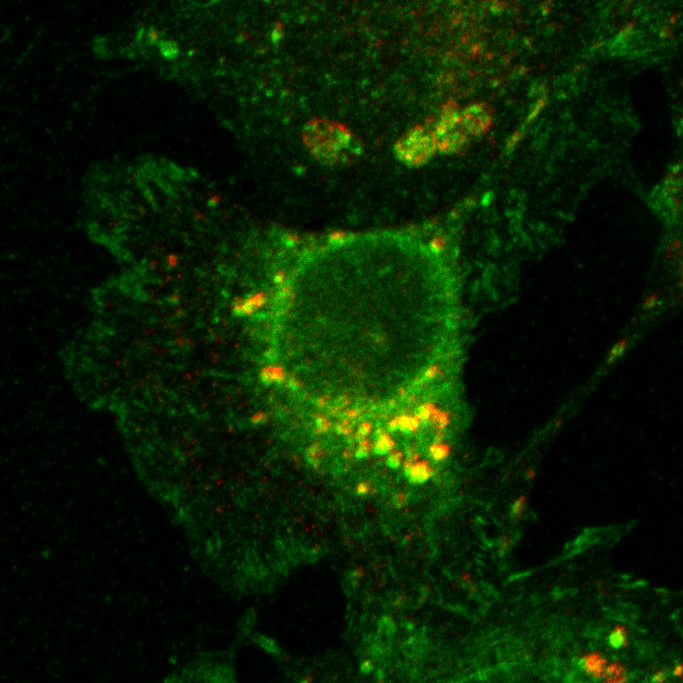

Supplement: Supplementary file 7 — Source data Fig. 4 [file 44319_2024_300_MOESM7_ESM.zip › Figure 4/4K/USP1246-dKO BafA1/Merge.tif]

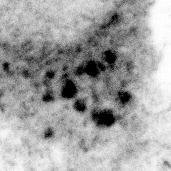

Supplement: Supplementary file 7 — Source data Fig. 4 [file 44319_2024_300_MOESM7_ESM.zip › Figure 4/4K/USP1246-dKO BafA1/Zoom in/Itgb1.png]

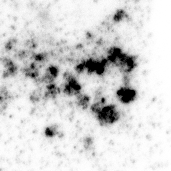

Supplement: Supplementary file 7 — Source data Fig. 4 [file 44319_2024_300_MOESM7_ESM.zip › Figure 4/4K/USP1246-dKO BafA1/Zoom in/Lamp1.png]

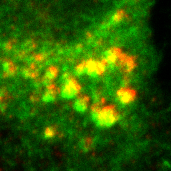

Supplement: Supplementary file 7 — Source data Fig. 4 [file 44319_2024_300_MOESM7_ESM.zip › Figure 4/4K/USP1246-dKO BafA1/Zoom in/merge.png]

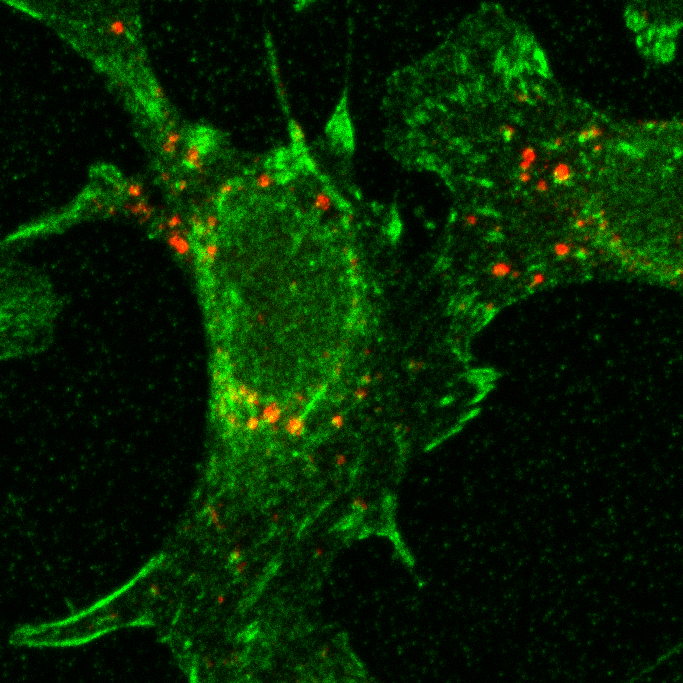

Supplement: Supplementary file 7 — Source data Fig. 4 [file 44319_2024_300_MOESM7_ESM.zip › Figure 4/4K/USP1246-dKO DMSO/Merge.tif]

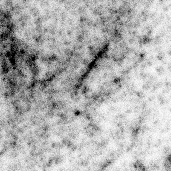

Supplement: Supplementary file 7 — Source data Fig. 4 [file 44319_2024_300_MOESM7_ESM.zip › Figure 4/4K/USP1246-dKO DMSO/Zoom in/Itgb1.png]

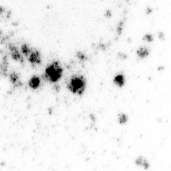

Supplement: Supplementary file 7 — Source data Fig. 4 [file 44319_2024_300_MOESM7_ESM.zip › Figure 4/4K/USP1246-dKO DMSO/Zoom in/Lamp1.png]

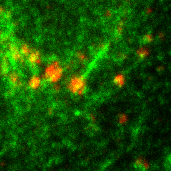

Supplement: Supplementary file 7 — Source data Fig. 4 [file 44319_2024_300_MOESM7_ESM.zip › Figure 4/4K/USP1246-dKO DMSO/Zoom in/merge.png]

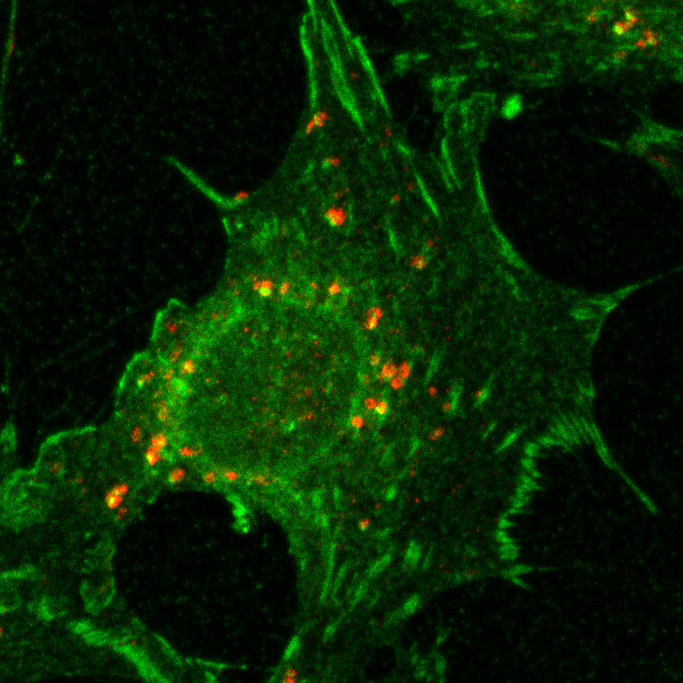

Supplement: Supplementary file 7 — Source data Fig. 4 [file 44319_2024_300_MOESM7_ESM.zip › Figure 4/4K/WT BafA1/Merge.tif]

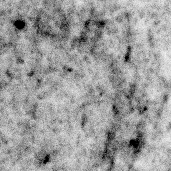

Supplement: Supplementary file 7 — Source data Fig. 4 [file 44319_2024_300_MOESM7_ESM.zip › Figure 4/4K/WT BafA1/Zoom in/Itgb1.png]

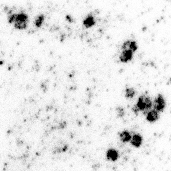

Supplement: Supplementary file 7 — Source data Fig. 4 [file 44319_2024_300_MOESM7_ESM.zip › Figure 4/4K/WT BafA1/Zoom in/Lamp1.png]

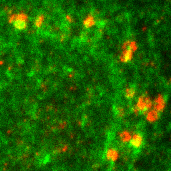

Supplement: Supplementary file 7 — Source data Fig. 4 [file 44319_2024_300_MOESM7_ESM.zip › Figure 4/4K/WT BafA1/Zoom in/merge.png]

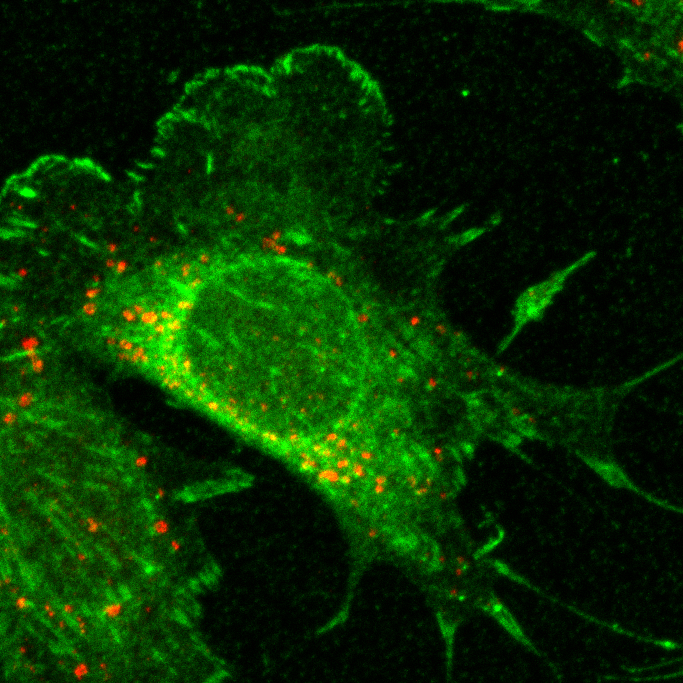

Supplement: Supplementary file 7 — Source data Fig. 4 [file 44319_2024_300_MOESM7_ESM.zip › Figure 4/4K/WT DMSO/Merge.tif]

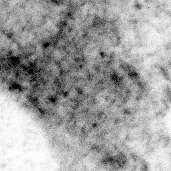

Supplement: Supplementary file 7 — Source data Fig. 4 [file 44319_2024_300_MOESM7_ESM.zip › Figure 4/4K/WT DMSO/ZOOM IN/Itgb1.png]

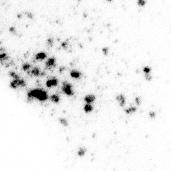

Supplement: Supplementary file 7 — Source data Fig. 4 [file 44319_2024_300_MOESM7_ESM.zip › Figure 4/4K/WT DMSO/ZOOM IN/Lamp1.png]

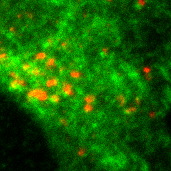

Supplement: Supplementary file 7 — Source data Fig. 4 [file 44319_2024_300_MOESM7_ESM.zip › Figure 4/4K/WT DMSO/ZOOM IN/merge.png]

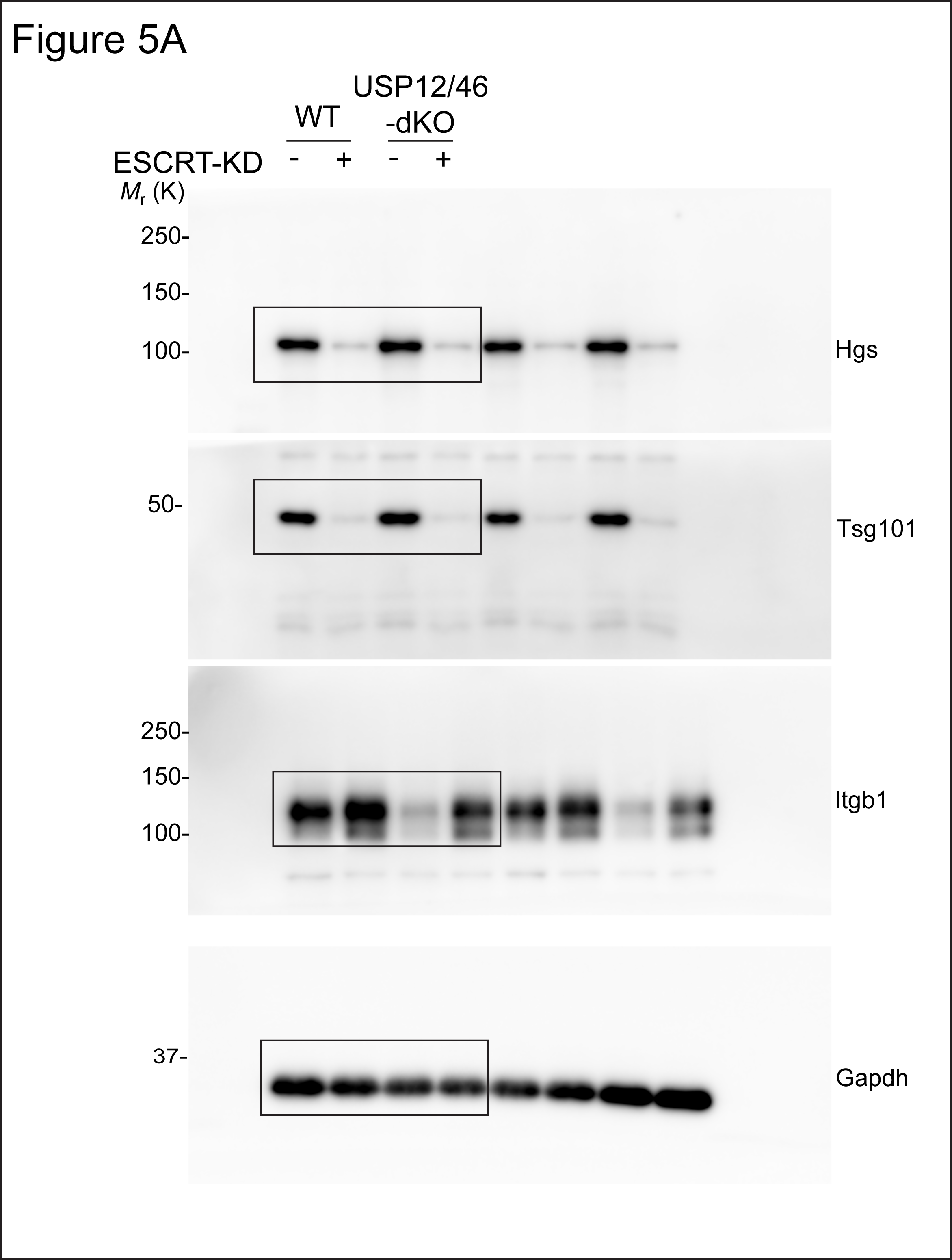

Supplement: Supplementary file 8 — Source data Fig. 5 [file 44319_2024_300_MOESM8_ESM.zip › Figure 5/5A/Figure 5A.tif]

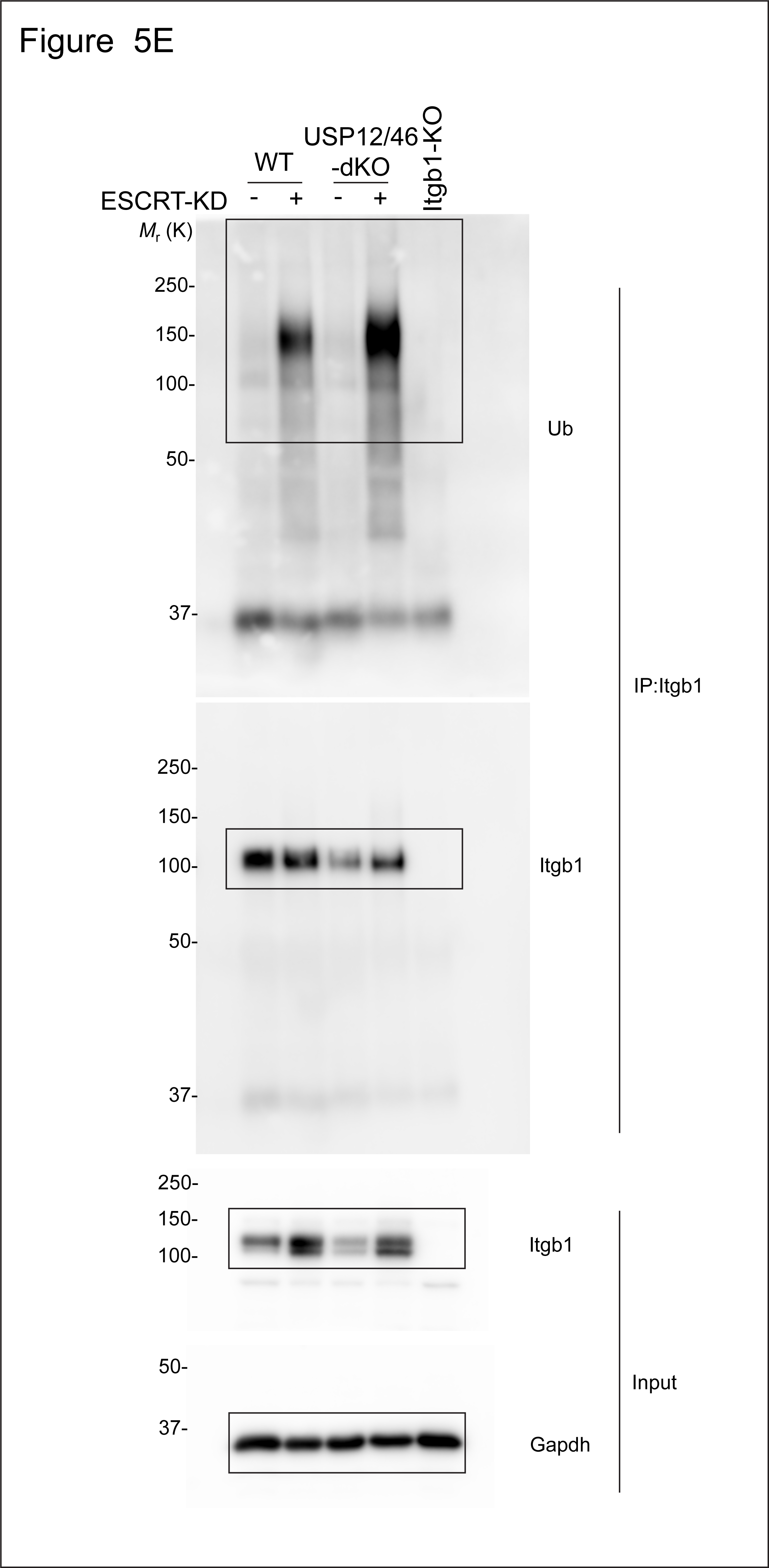

Supplement: Supplementary file 8 — Source data Fig. 5 [file 44319_2024_300_MOESM8_ESM.zip › Figure 5/5E/Figure 5E.tif]

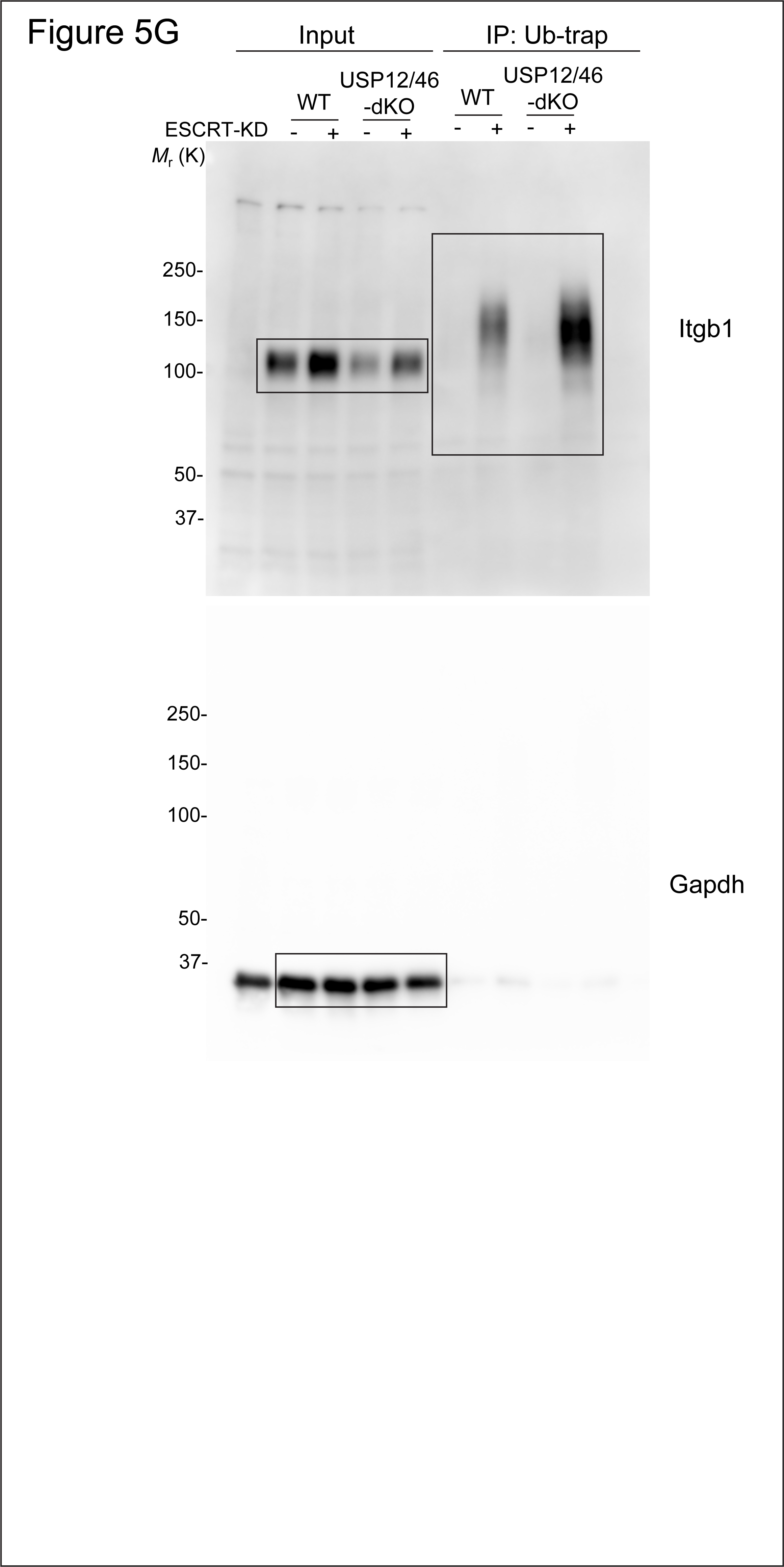

Supplement: Supplementary file 8 — Source data Fig. 5 [file 44319_2024_300_MOESM8_ESM.zip › Figure 5/5G/Figure 5G.tif]

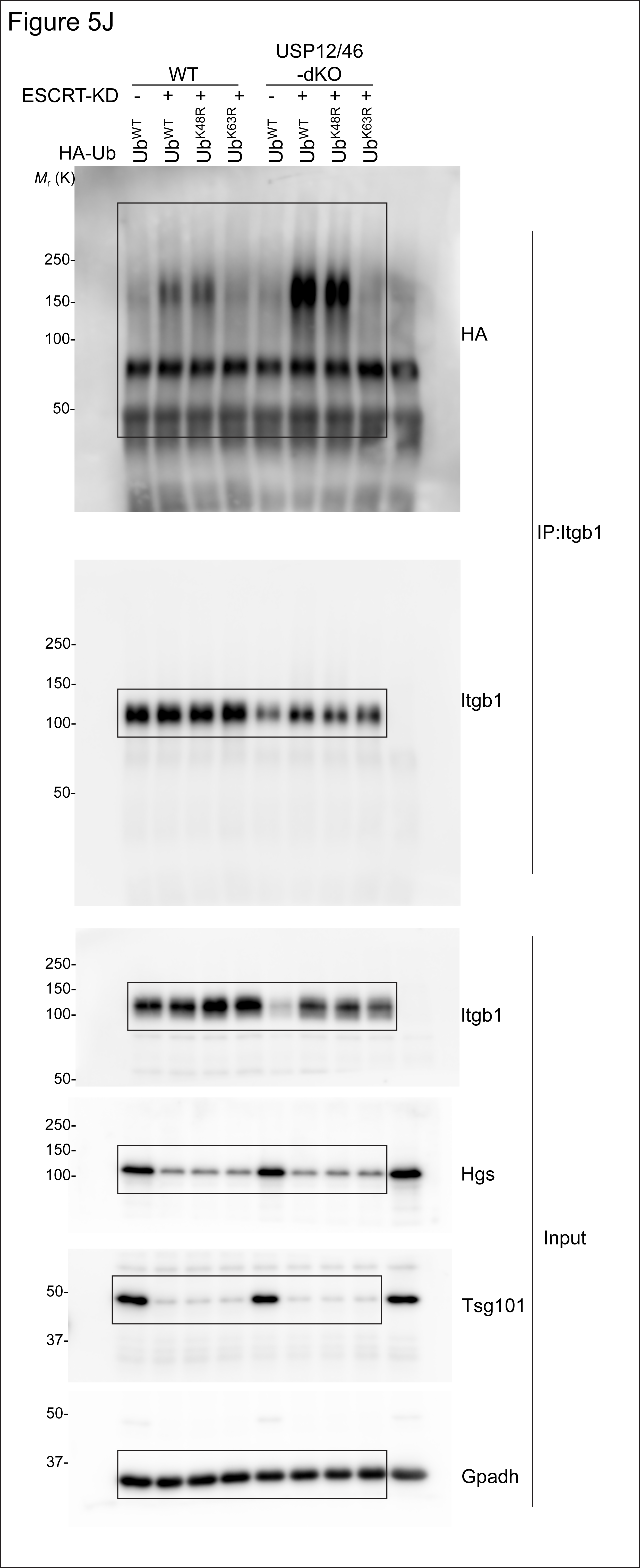

Supplement: Supplementary file 8 — Source data Fig. 5 [file 44319_2024_300_MOESM8_ESM.zip › Figure 5/5J/Figure 5J.tif]

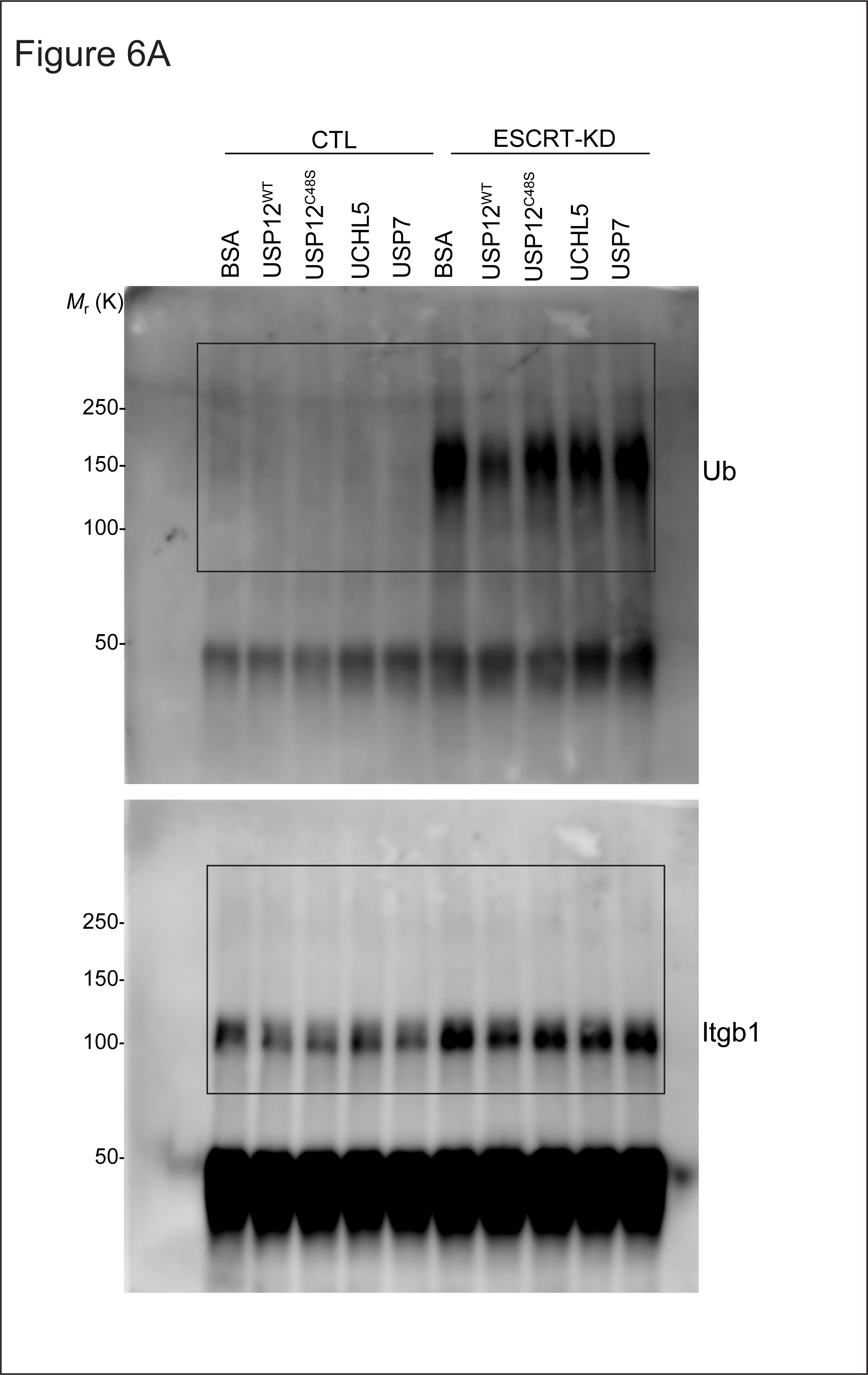

Supplement: Supplementary file 9 — Source data Fig. 6 [file 44319_2024_300_MOESM9_ESM.zip › Figure 6/6A/Figure 6A.tif]

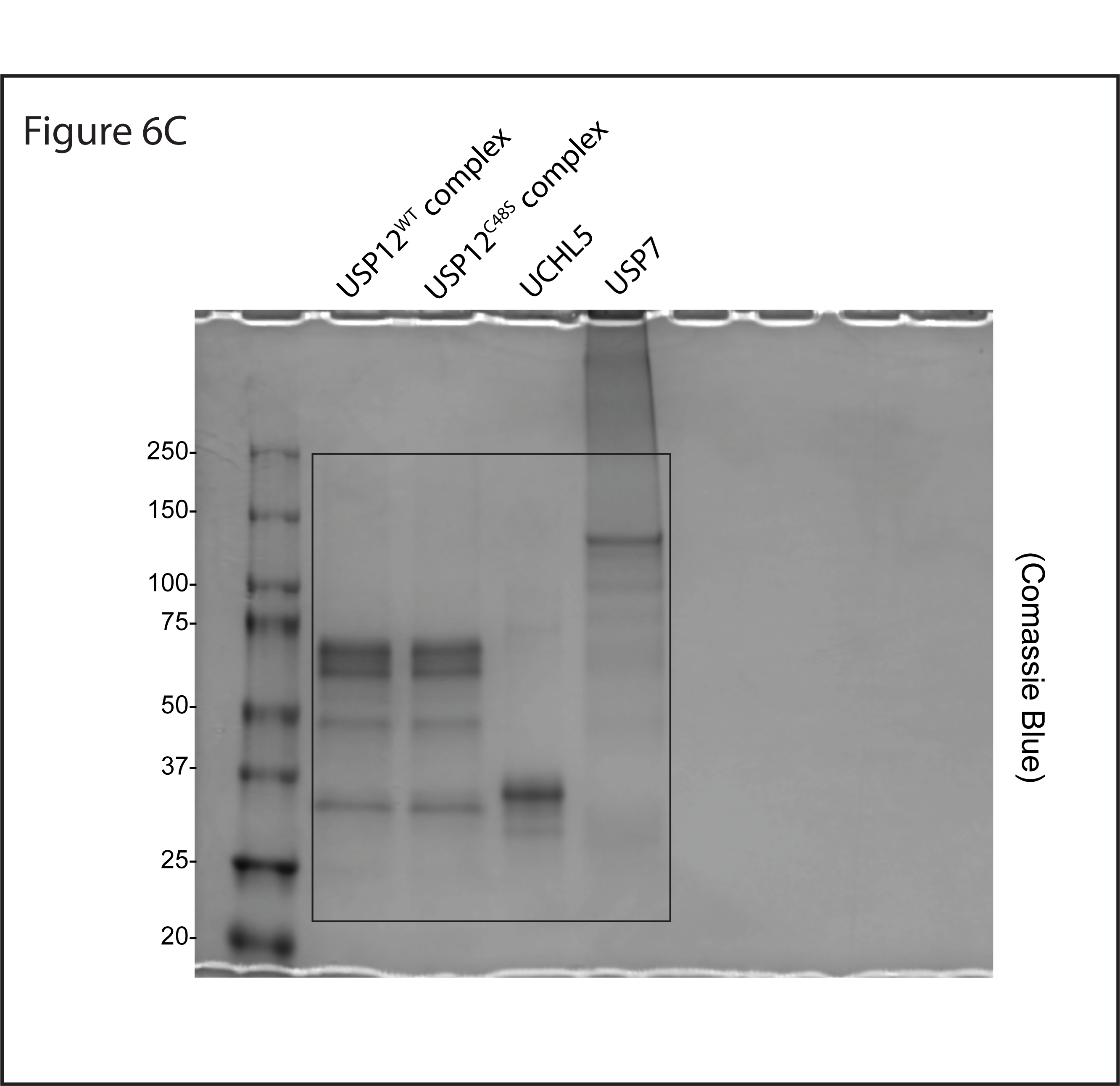

Supplement: Supplementary file 9 — Source data Fig. 6 [file 44319_2024_300_MOESM9_ESM.zip › Figure 6/6C/Figure 6C.tif]

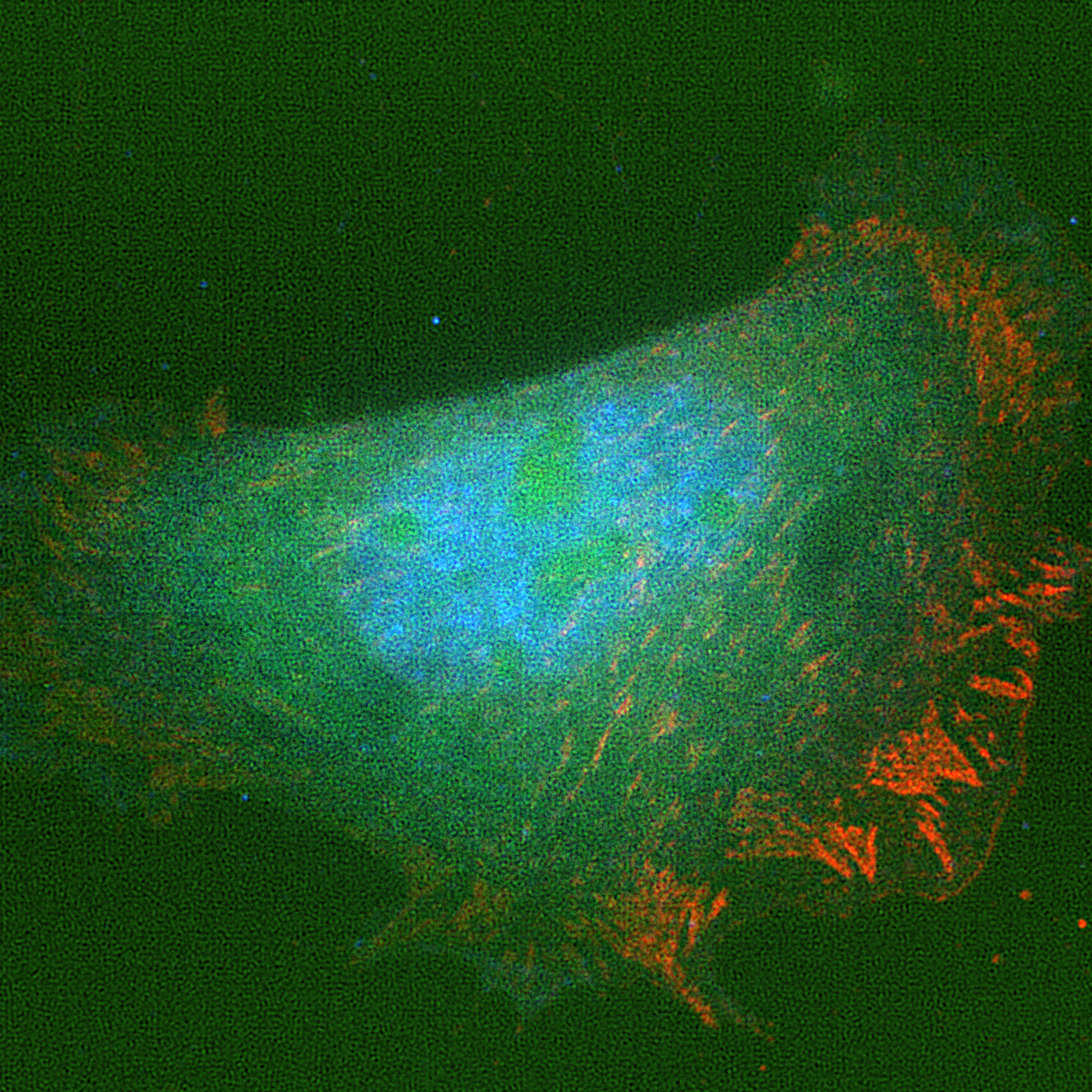

Supplement: Supplementary file 9 — Source data Fig. 6 [file 44319_2024_300_MOESM9_ESM.zip › Figure 6/6D/CTL/Merge.tif]

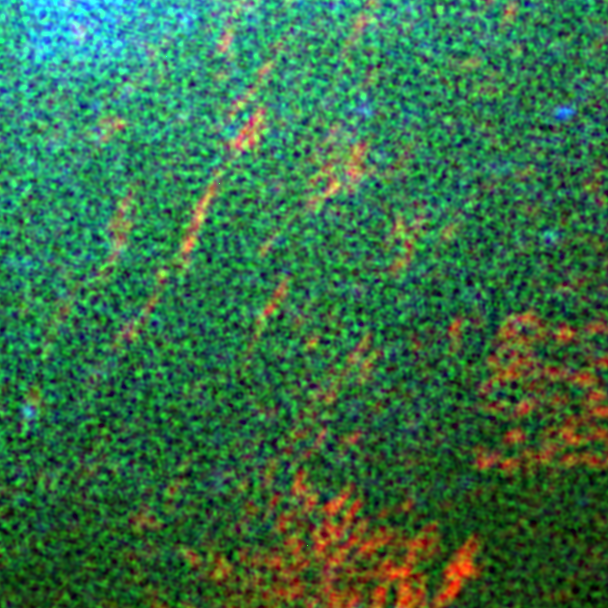

Supplement: Supplementary file 9 — Source data Fig. 6 [file 44319_2024_300_MOESM9_ESM.zip › Figure 6/6D/CTL/Zoom in/Merge.tif]

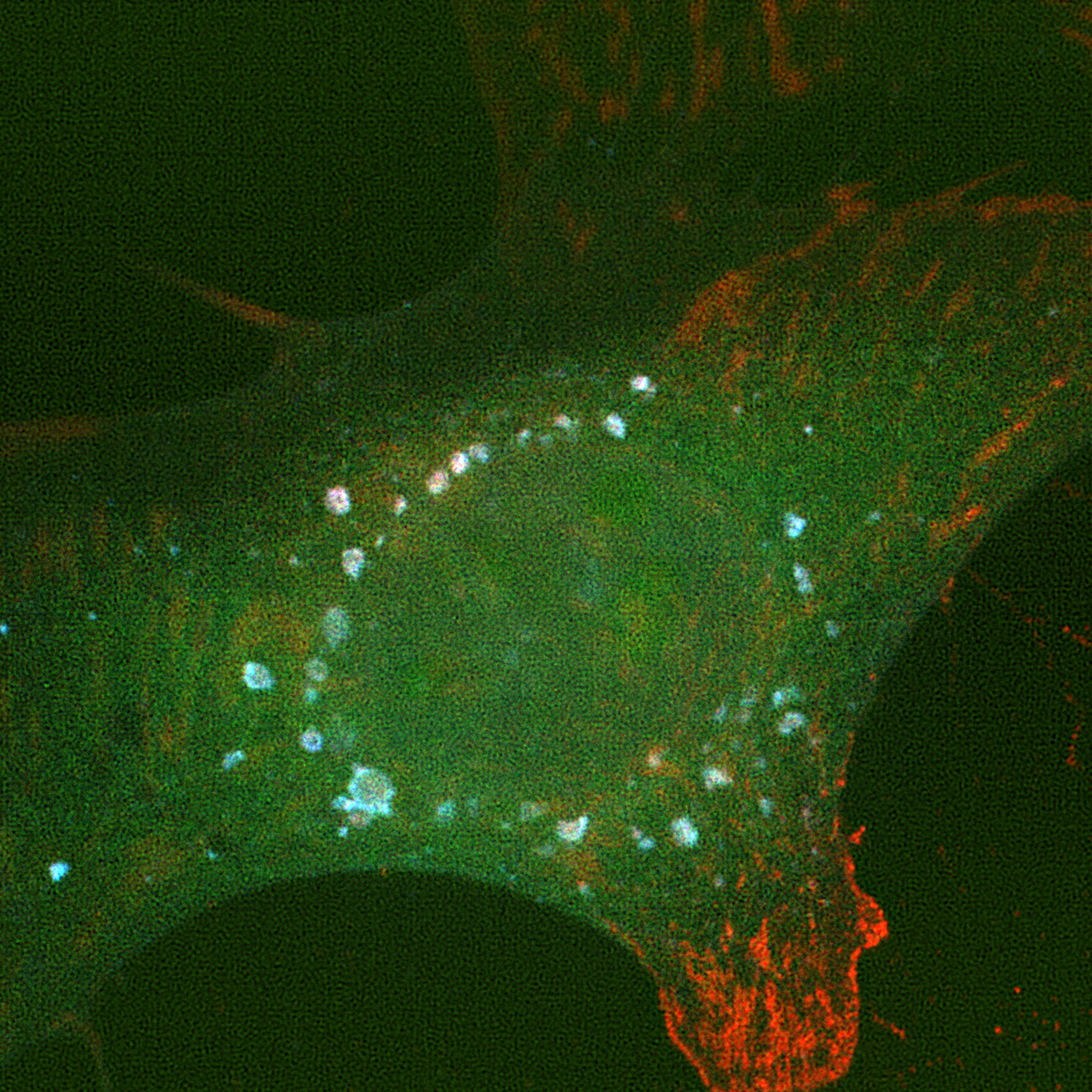

Supplement: Supplementary file 9 — Source data Fig. 6 [file 44319_2024_300_MOESM9_ESM.zip › Figure 6/6D/ESCRT-KD/Merge.tif]

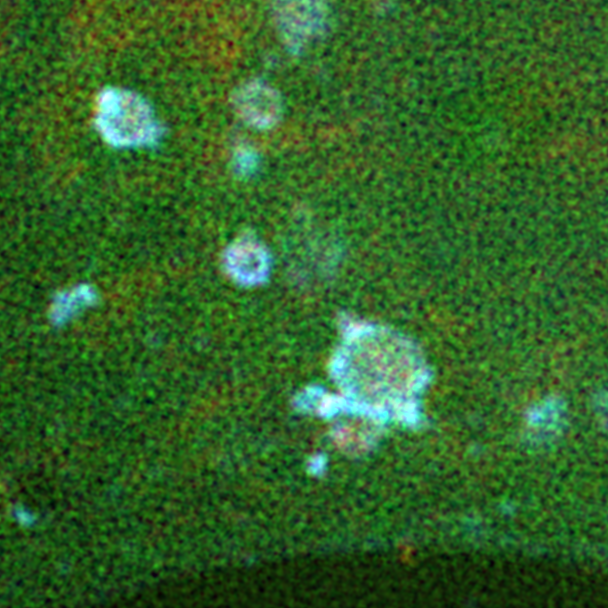

Supplement: Supplementary file 9 — Source data Fig. 6 [file 44319_2024_300_MOESM9_ESM.zip › Figure 6/6D/ESCRT-KD/Zoom in/Merge.tif]

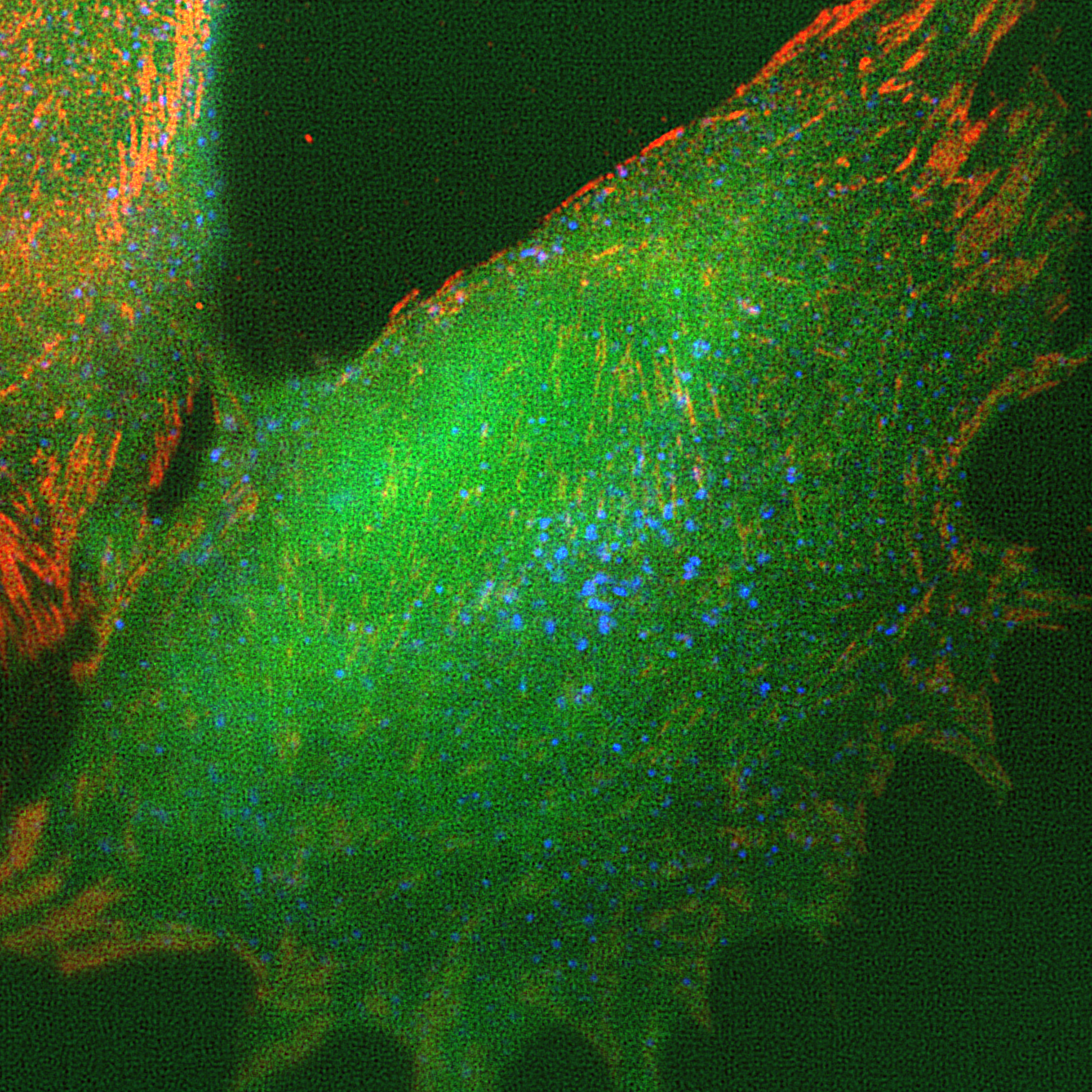

Supplement: Supplementary file 9 — Source data Fig. 6 [file 44319_2024_300_MOESM9_ESM.zip › Figure 6/6H/CTL/Merge.tif]

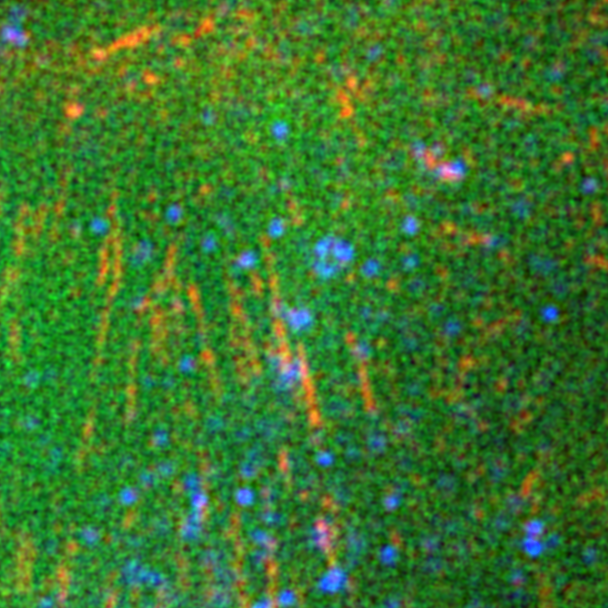

Supplement: Supplementary file 9 — Source data Fig. 6 [file 44319_2024_300_MOESM9_ESM.zip › Figure 6/6H/CTL/Zoom in/Merge.tif]

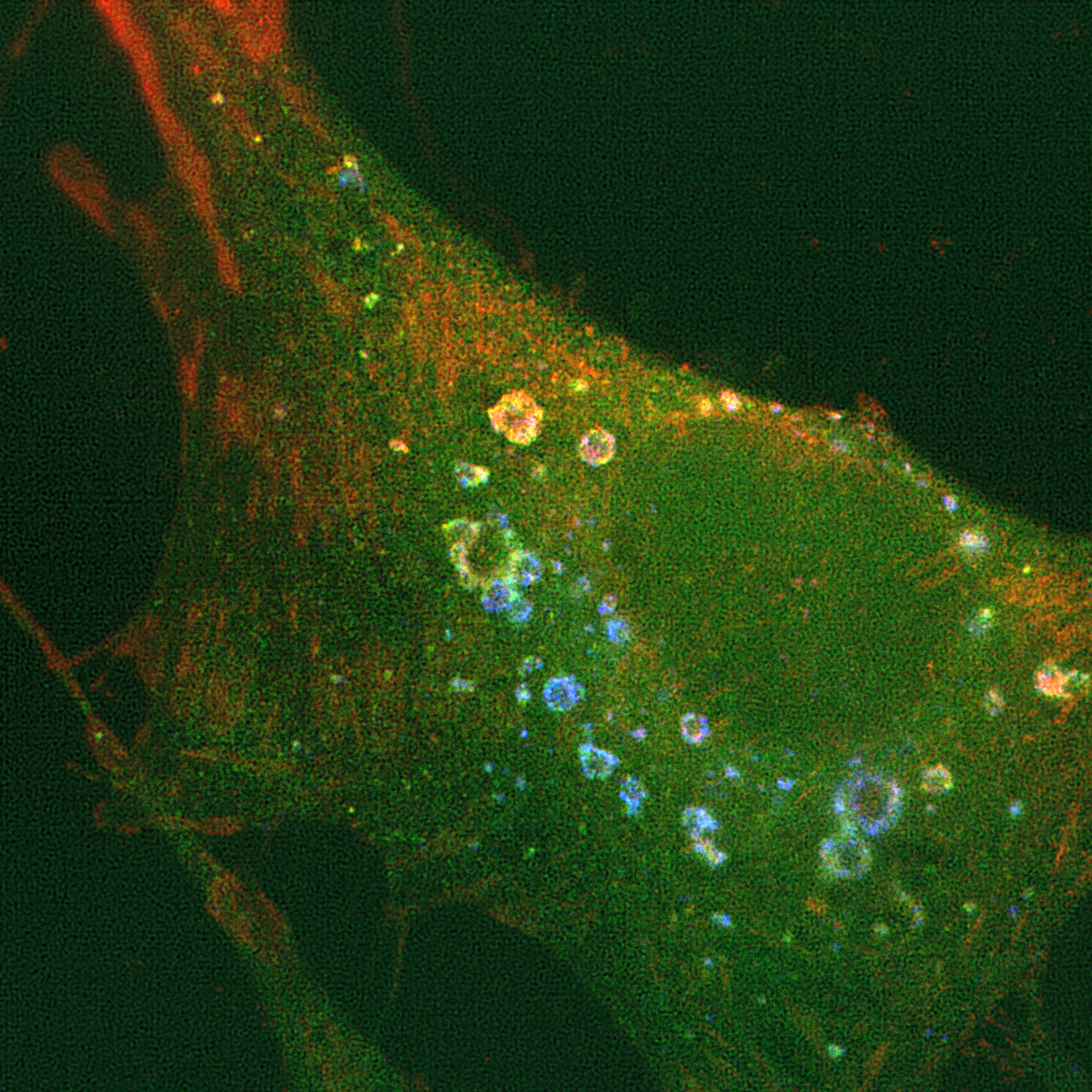

Supplement: Supplementary file 9 — Source data Fig. 6 [file 44319_2024_300_MOESM9_ESM.zip › Figure 6/6H/ESCRT-KD/Merge.tif]

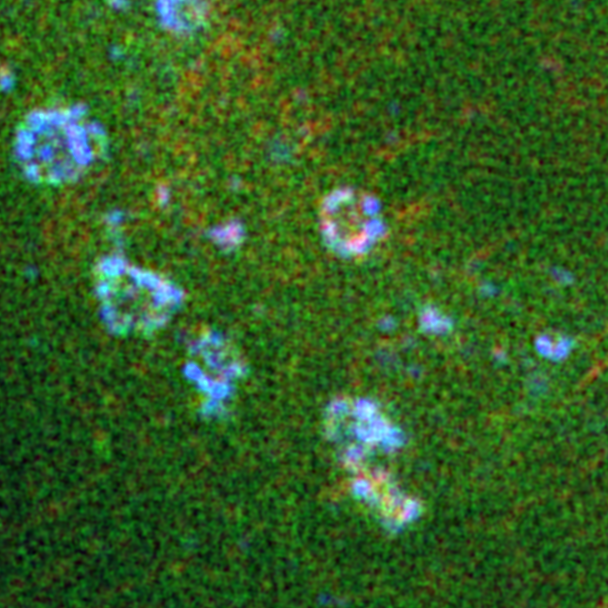

Supplement: Supplementary file 9 — Source data Fig. 6 [file 44319_2024_300_MOESM9_ESM.zip › Figure 6/6H/ESCRT-KD/Zoom in/Merge.tif]

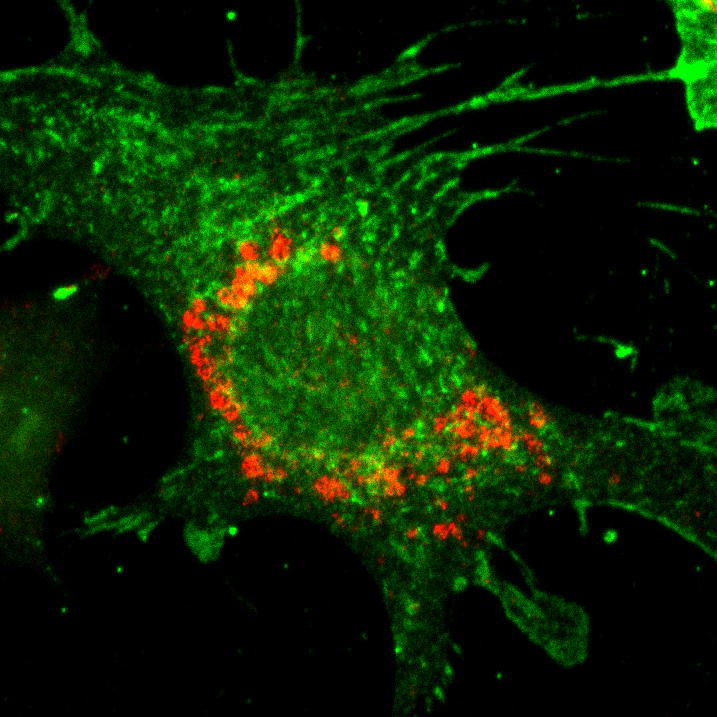

Supplement: Supplementary file 10 — Source data Fig. 7 [file 44319_2024_300_MOESM10_ESM.zip › Figure 7/7B/USP1246 dKO a5KRb1KR/Merge.tif]

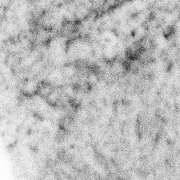

Supplement: Supplementary file 10 — Source data Fig. 7 [file 44319_2024_300_MOESM10_ESM.zip › Figure 7/7B/USP1246 dKO a5KRb1KR/Zoom in/h-Itga5.png]

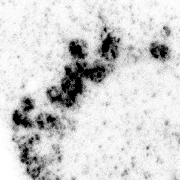

Supplement: Supplementary file 10 — Source data Fig. 7 [file 44319_2024_300_MOESM10_ESM.zip › Figure 7/7B/USP1246 dKO a5KRb1KR/Zoom in/Lamp1.png]

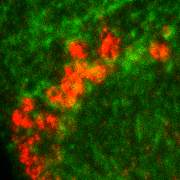

Supplement: Supplementary file 10 — Source data Fig. 7 [file 44319_2024_300_MOESM10_ESM.zip › Figure 7/7B/USP1246 dKO a5KRb1KR/Zoom in/merge.png]

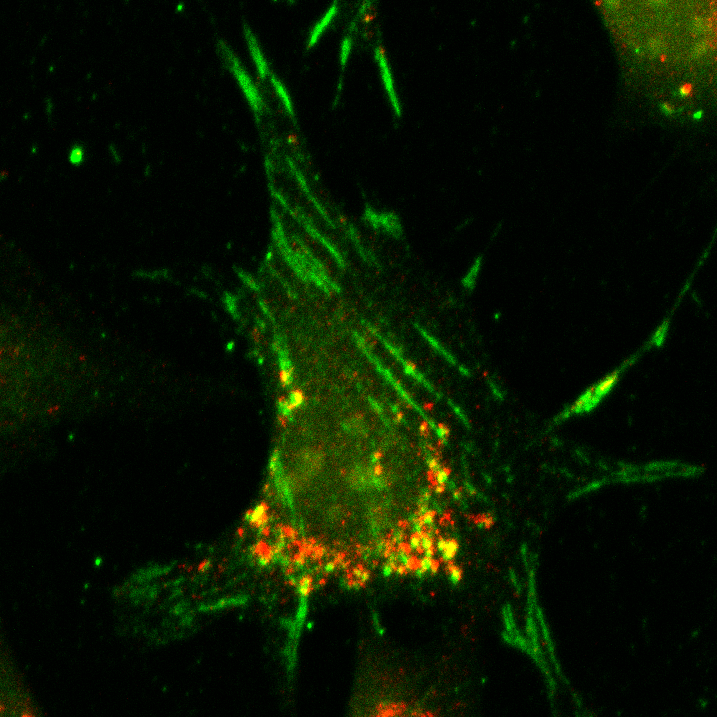

Supplement: Supplementary file 10 — Source data Fig. 7 [file 44319_2024_300_MOESM10_ESM.zip › Figure 7/7B/USP1246 dKO a5WTb1WT/merge.tif]

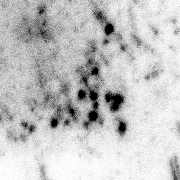

Supplement: Supplementary file 10 — Source data Fig. 7 [file 44319_2024_300_MOESM10_ESM.zip › Figure 7/7B/USP1246 dKO a5WTb1WT/Zoom in/h-Itga5.png]

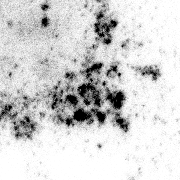

Supplement: Supplementary file 10 — Source data Fig. 7 [file 44319_2024_300_MOESM10_ESM.zip › Figure 7/7B/USP1246 dKO a5WTb1WT/Zoom in/Lamp1.png]

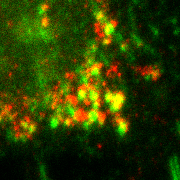

Supplement: Supplementary file 10 — Source data Fig. 7 [file 44319_2024_300_MOESM10_ESM.zip › Figure 7/7B/USP1246 dKO a5WTb1WT/Zoom in/merge.png]

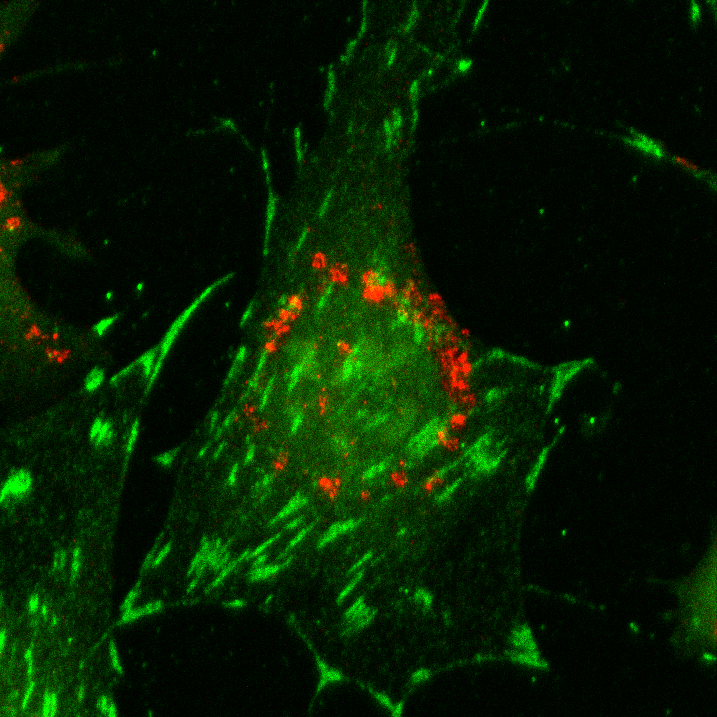

Supplement: Supplementary file 10 — Source data Fig. 7 [file 44319_2024_300_MOESM10_ESM.zip › Figure 7/7B/WT a5KRb1KR/Merge.tif]

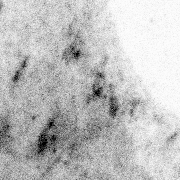

Supplement: Supplementary file 10 — Source data Fig. 7 [file 44319_2024_300_MOESM10_ESM.zip › Figure 7/7B/WT a5KRb1KR/Zoom in/h-Itga5.png]

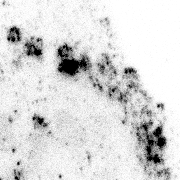

Supplement: Supplementary file 10 — Source data Fig. 7 [file 44319_2024_300_MOESM10_ESM.zip › Figure 7/7B/WT a5KRb1KR/Zoom in/Lamp1.png]

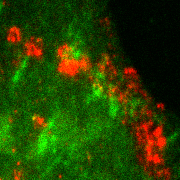

Supplement: Supplementary file 10 — Source data Fig. 7 [file 44319_2024_300_MOESM10_ESM.zip › Figure 7/7B/WT a5KRb1KR/Zoom in/merge.png]

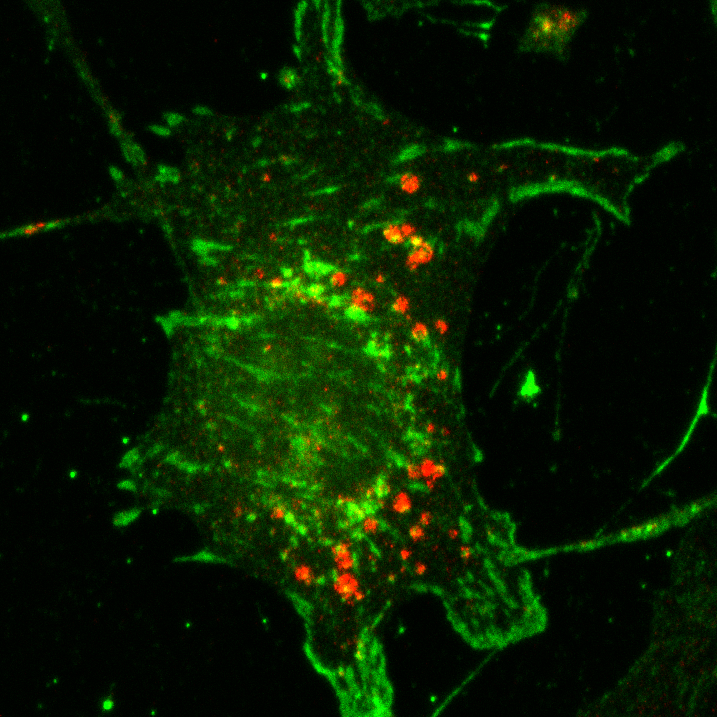

Supplement: Supplementary file 10 — Source data Fig. 7 [file 44319_2024_300_MOESM10_ESM.zip › Figure 7/7B/WT a5WTb1WT/Merge.tif]

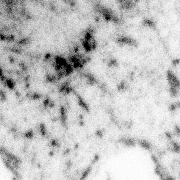

Supplement: Supplementary file 10 — Source data Fig. 7 [file 44319_2024_300_MOESM10_ESM.zip › Figure 7/7B/WT a5WTb1WT/Zoom in/h-Itga5.png]

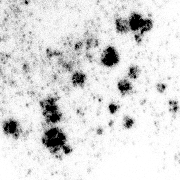

Supplement: Supplementary file 10 — Source data Fig. 7 [file 44319_2024_300_MOESM10_ESM.zip › Figure 7/7B/WT a5WTb1WT/Zoom in/Lamp1.png]

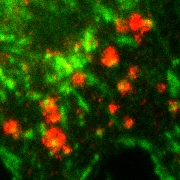

Supplement: Supplementary file 10 — Source data Fig. 7 [file 44319_2024_300_MOESM10_ESM.zip › Figure 7/7B/WT a5WTb1WT/Zoom in/merge.png]

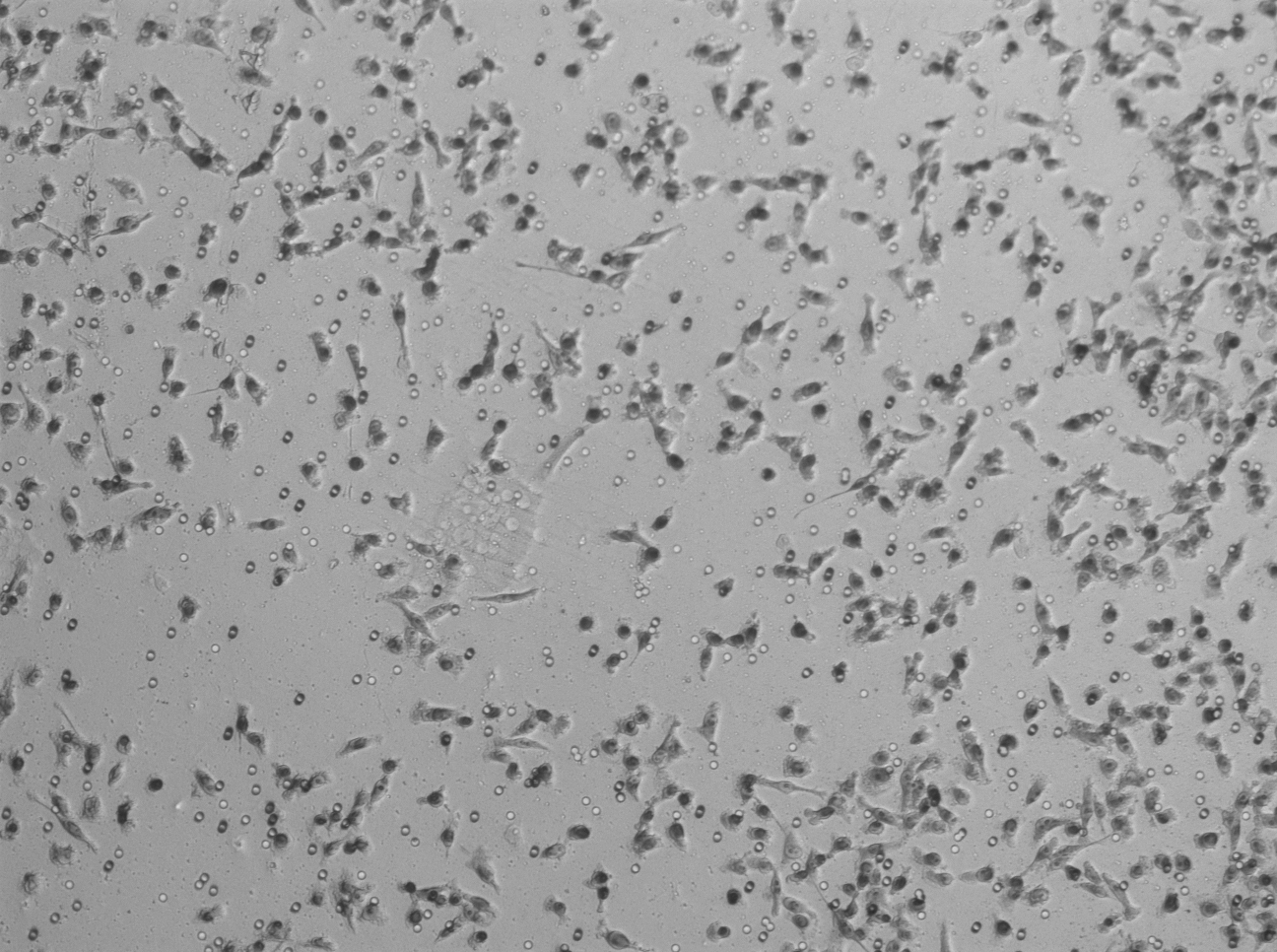

Supplement: Supplementary file 11 — Source data Fig. 8 [file 44319_2024_300_MOESM11_ESM.zip › Figure 8/8D/USP1246 dKO cl1.tif]

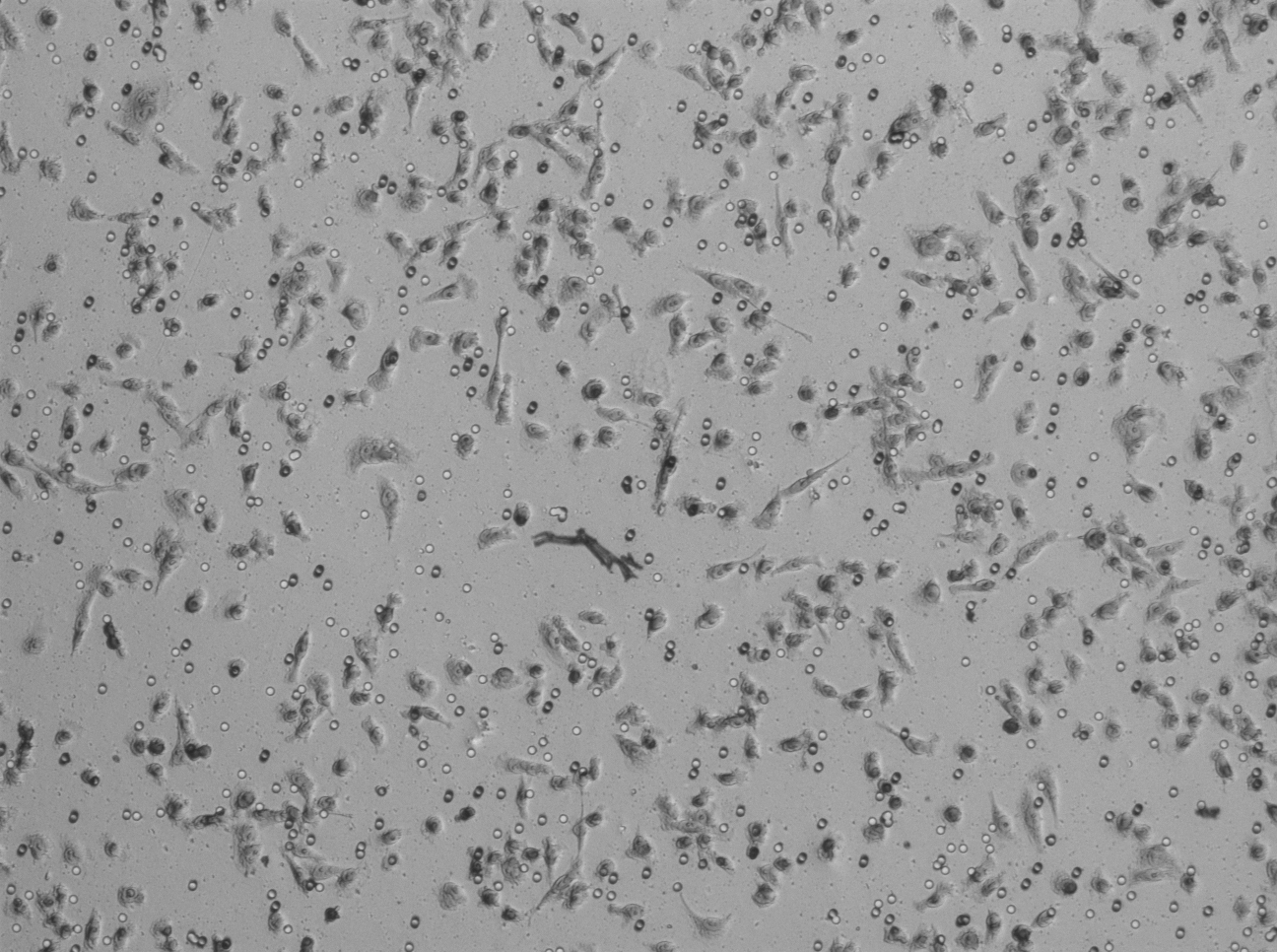

Supplement: Supplementary file 11 — Source data Fig. 8 [file 44319_2024_300_MOESM11_ESM.zip › Figure 8/8D/USP1246 dKO cl2.tif]

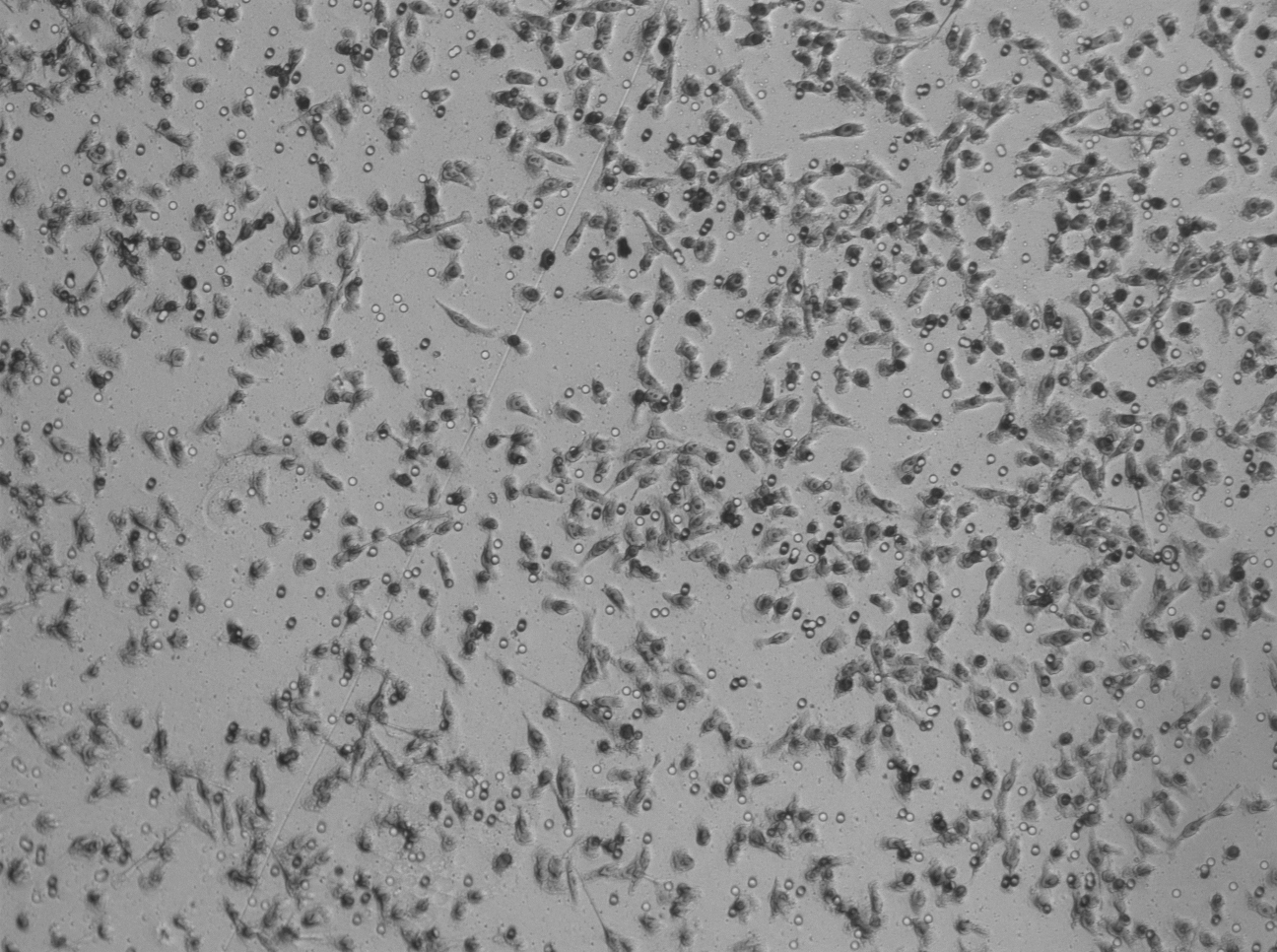

Supplement: Supplementary file 11 — Source data Fig. 8 [file 44319_2024_300_MOESM11_ESM.zip › Figure 8/8D/WT.tif]

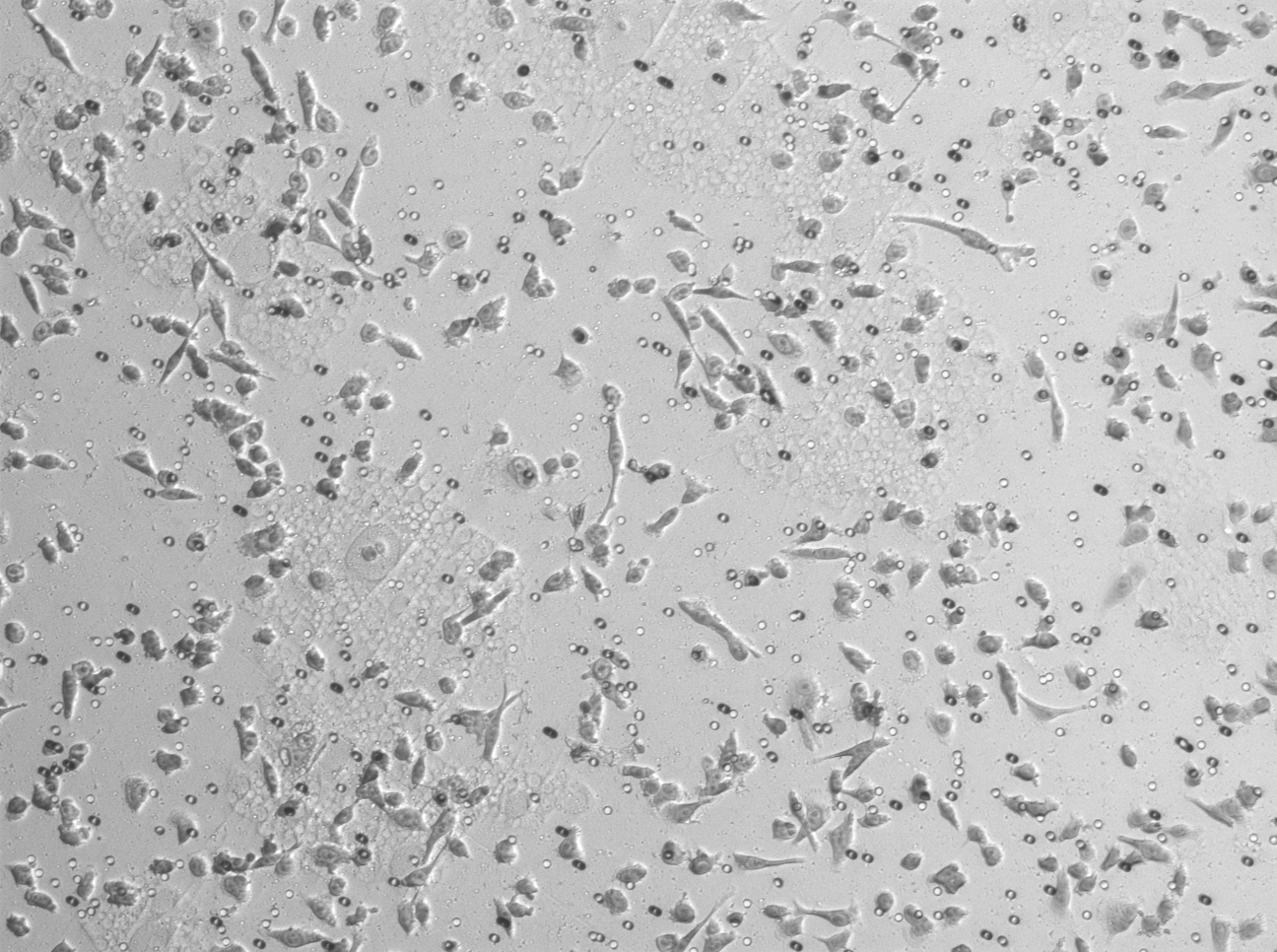

Supplement: Supplementary file 11 — Source data Fig. 8 [file 44319_2024_300_MOESM11_ESM.zip › Figure 8/8F/USP1246 dKO cl1.tif]

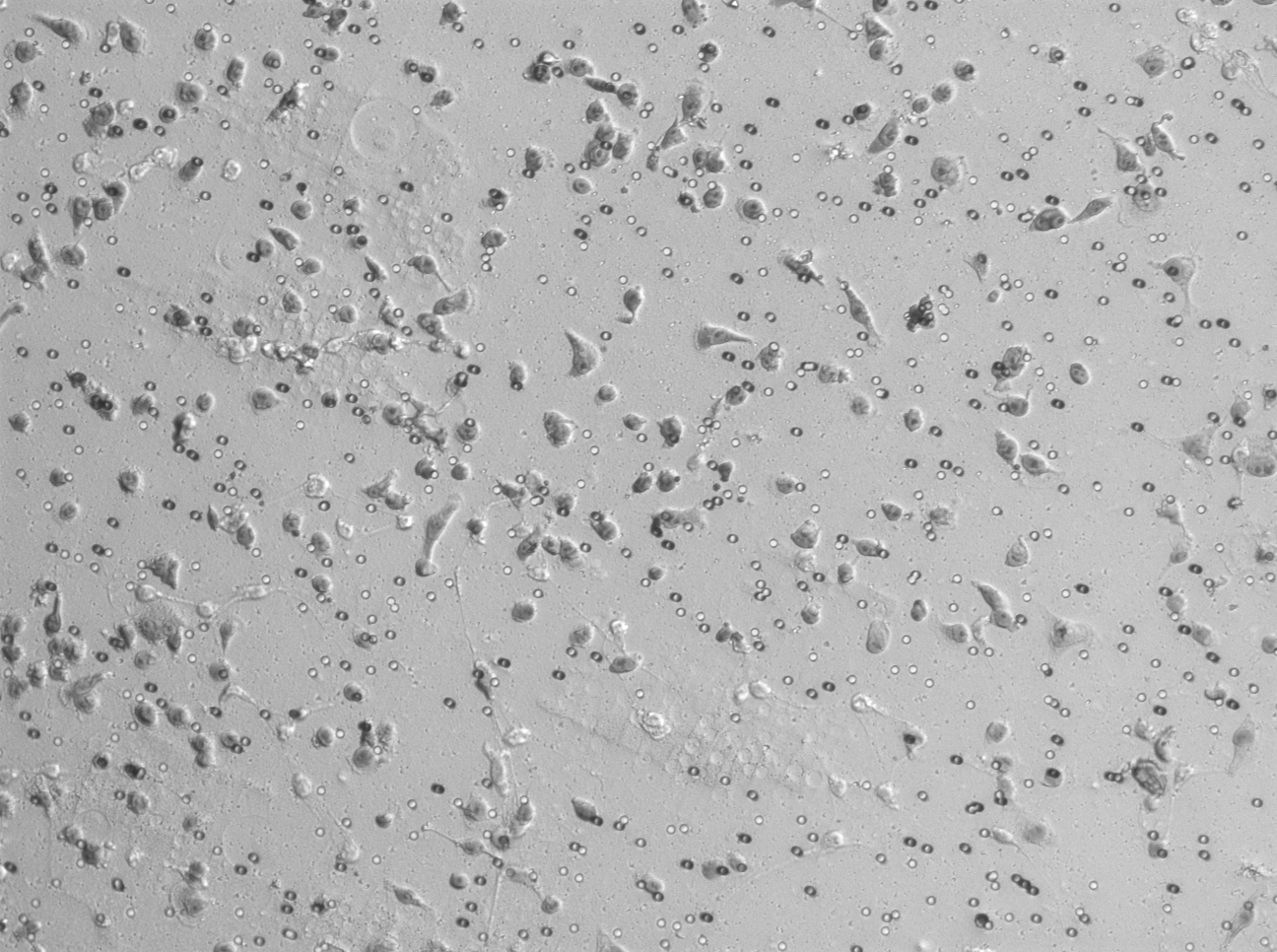

Supplement: Supplementary file 11 — Source data Fig. 8 [file 44319_2024_300_MOESM11_ESM.zip › Figure 8/8F/USP1246 dKO cl2.tif]

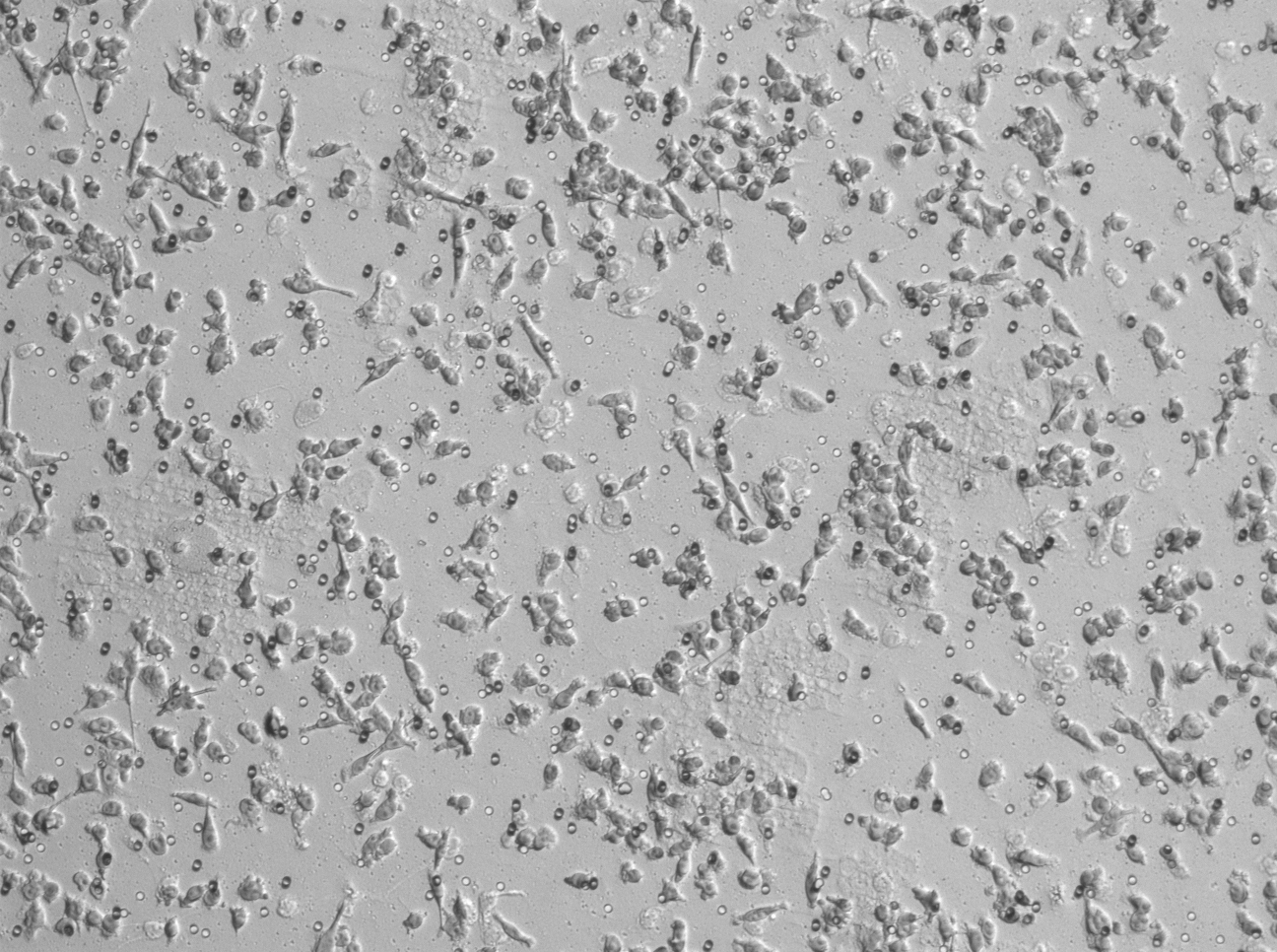

Supplement: Supplementary file 11 — Source data Fig. 8 [file 44319_2024_300_MOESM11_ESM.zip › Figure 8/8F/WT.tif]
