# Supplementary material for: The USP12/46 deubiquitinases protect integrins from ESCRT-mediated lysosomal degradation
Source: EMBO Rep. 2024 Nov 6;25(12):5687–718. doi: 10.1038/s44319-024-00300-9 (PMC11624278; doi:10.1038/s44319-024-00300-9)
Supplement: Supplementary file 12 — Expanded View Figures [file 44319_2024_300_MOESM12_ESM.pdf]

Expanded View Figures

Figure EV1. Related to Fig. 1. The effect of USP9X-KD on integrin levels.

(**A–H**) WB and densitometric quantification of Itga5 and Itgb1 protein levels (**A**, **C**, **E**, **G**) and flow cytometry analysis of Itga5 and Itgb1 surface levels (**B**, **D**, **F**, **H**) in mouse fibroblasts (**A**, **B**), Hela (**C**, **D**), RPE-1 (**E**, **F**), and MDA-MB-231 cells (**G**, **H**) treated with control non-targeting siRNA (CTL) or siRNAs targeting USP9X (USP9X-KD). Cells were cultured overnight in DMEM with 10% serum or serum-replacement medium. Gapdh served as a loading control. Statistical analysis was carried out by paired *t*-test. In (**A**), statistical significance was tested between the CTL and USP9X-KD groups with or without serum (for Itga5, $P = 0.2973$ and 0.3151, respectively; for Itgb1, $P = 0.1435$ and 0.9821, respectively). In (**B**), statistical significance was tested between the CTL and USP9X-KD groups with or without serum (for Itga5, $P = 0.2412$ and 0.0346, respectively; for Itgb1, $P = 0.6668$ and 0.4548, respectively). In (**C**), statistical significance was tested between the CTL and USP9X-KD groups with or without serum (for Itga5, $P = 0.0573$ and 0.0321, respectively; for Itgb1, $P = 0.8148$ and 0.1067, respectively). In (**D**), statistical significance was tested between the CTL and USP9X-KD groups with or without serum (for Itga5, $P = 0.1340$ and 0.1064, respectively; for Itgb1, $P = 0.0409$ and 0.0622, respectively). In (**E**), statistical significance was tested between the CTL and USP9X-KD groups with or without serum (for Itga5, $P = 0.0110$ and 0.0457, respectively; for Itgb1, $P = 0.1110$ and 0.2666, respectively). In (**F**), statistical significance was tested between the CTL and USP9X-KD groups with or without serum (for Itga5, $P = 0.1336$ and 0.1802, respectively; for Itgb1, $P = 0.0086$ and 0.0620, respectively). In (**G**), statistical significance was tested between the CTL and USP9X-KD groups with or without serum (for Itga5, $P = 0.2786$ and 0.0450, respectively; for Itgb1, $P = 0.0271$ and 0.1004, respectively). In (**H**), statistical significance was tested between the CTL and USP9X-KD groups with or without serum (for Itga5, $P = 0.0117$ and 0.0001, respectively; for Itgb1, $P = 0.0986$ and 0.0954, respectively). *$P < 0.05$; **$P < 0.01$; ***$P < 0.001$; n.s. not significant. Data were shown as Mean ± SD, $n = 3$ independent experiments.

© The Author(s)

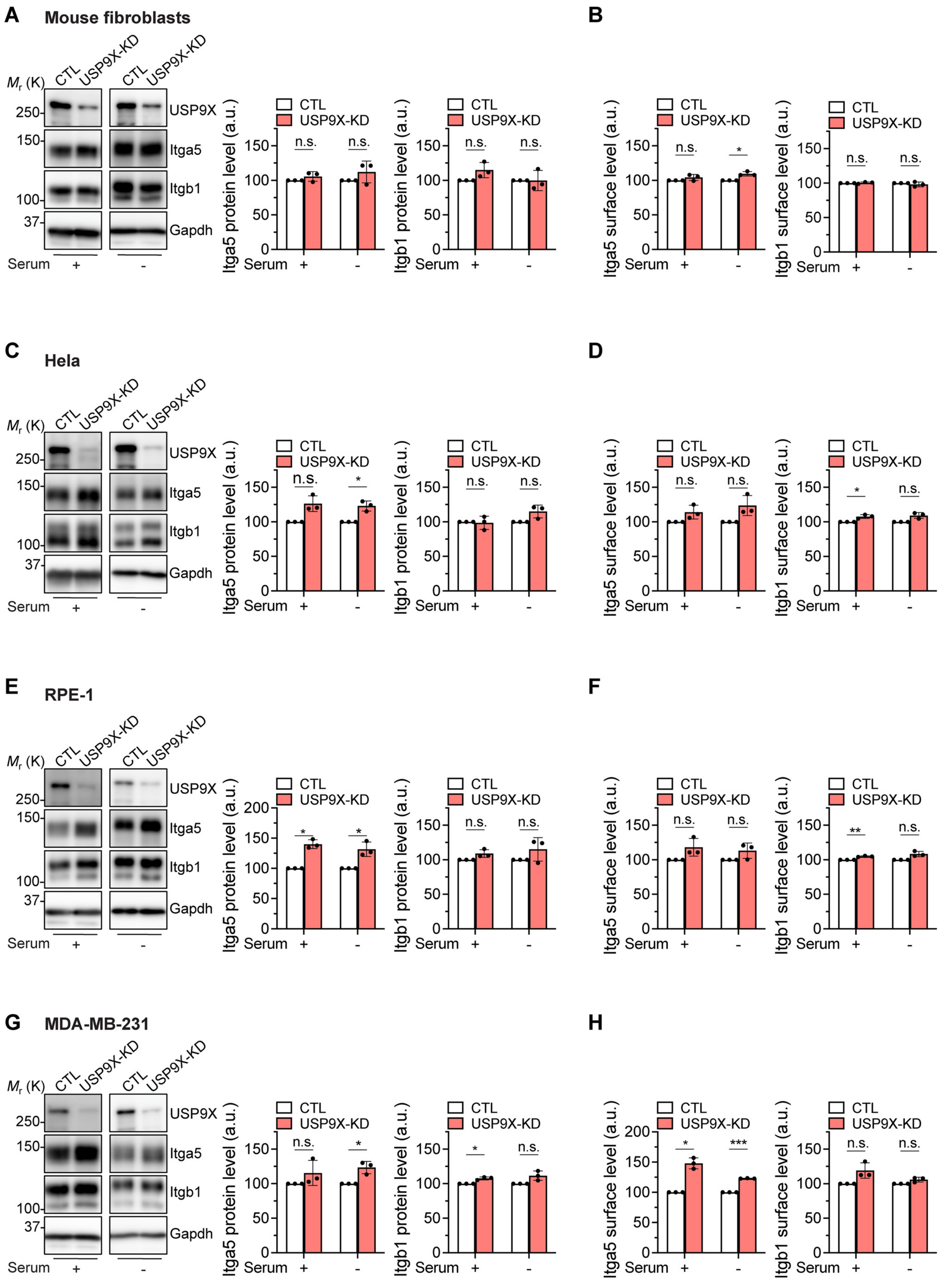
A Mouse fibroblasts
B
C Hela
D
E RPE-1
F
G MDA-MB-231
H
Mr (K)
CTL
USP9X-KD
USP9X
Itga5
Itgb1
Gapdh
Serum
Itga5 protein level (a.u.)
Itgb1 protein level (a.u.)
Itga5 surface level (a.u.)
Itgb1 surface level (a.u.)
n.s.

© The Author(s)

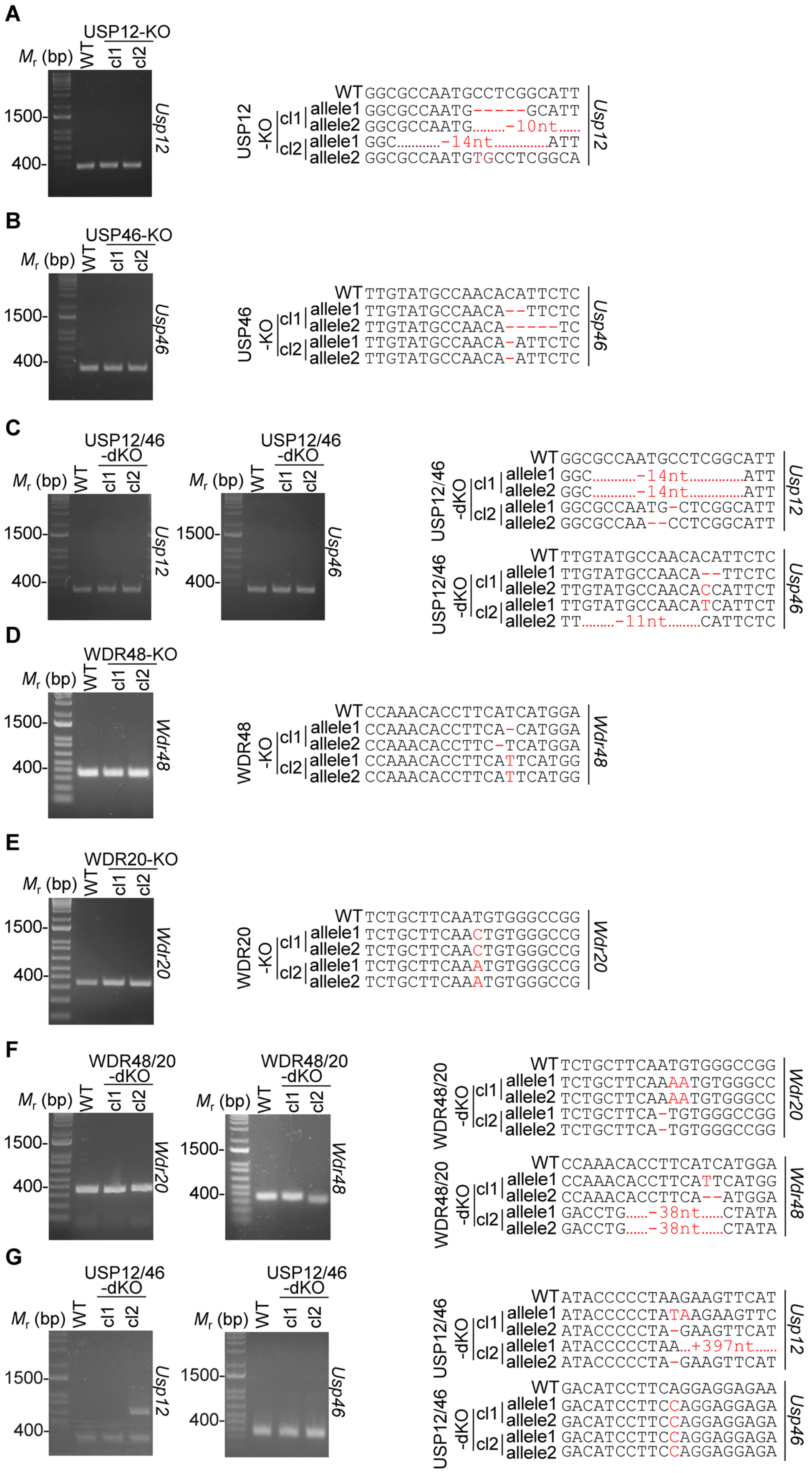
A
USP12-KO
M_r (bp) WT cl1 cl2
1500-
400-
Usp12
WT GGCGCCAATGCCTCGGCATT
USP12 -KO cl1 allele1 GGCGCCAATG-----GCATT
allele2 GGCGCCAATG.........-10nt......
cl2 allele1 GGC............-14nt...............ATT
allele2 GGCGCCAATGTGCCTCGGCA
B
USP46-KO
Usp46
WT TTGTATGCCAACACATTCTC
USP46 -KO cl1 allele1 TTGTATGCCAACA--TTCTC
allele2 TTGTATGCCAACA-----TC
cl2 allele1 TTGTATGCCAACA-ATTCTC
allele2 TTGTATGCCAACA-ATTCTC
C
USP12/46 -dKO
WT GGCGCCAATGCCTCGGCATT
cl1 allele1 GGC............-14nt...............ATT
allele2 GGC............-14nt...............ATT
cl2 allele1 GGCGCCAATG-CTCGGCATT
allele2 GGCGCCAA--CCTCGGCATT
WT TTGTATGCCAACACATTCTC
cl1 allele1 TTGTATGCCAACA--TTCTC
allele2 TTGTATGCCAACACCATTCT
cl2 allele1 TTGTATGCCAACATCATTCT
allele2 TT.........-11nt.........CATTCTC
D
WDR48-KO
Wdr48
WT CCAAACACCTTCATCATGGA
WDR48 -KO cl1 allele1 CCAAACACCTTCA-CATGGA
allele2 CCAAACACCTTC-TCATGGA
cl2 allele1 CCAAACACCTTCATTCATGG
allele2 CCAAACACCTTCATTCATGG
E
WDR20-KO
Wdr20
WT TCTGCTTCAATGTGGGCCGG
WDR20 -KO cl1 allele1 TCTGCTTCAACTGTGGGCCG
allele2 TCTGCTTCAACTGTGGGCCG
cl2 allele1 TCTGCTTCAAATGTGGGCCG
allele2 TCTGCTTCAAATGTGGGCCG
F
WDR48/20 -dKO
WT TCTGCTTCAATGTGGGCCGG
cl1 allele1 TCTGCTTCAAAATGTGGGCC
allele2 TCTGCTTCAAAATGTGGGCC
cl2 allele1 TCTGCTTCA-TGTGGGCCGG
allele2 TCTGCTTCA-TGTGGGCCGG
WT CCAAACACCTTCATCATGGA
cl1 allele1 CCAAACACCTTCATTCATGG
allele2 CCAAACACCTTCA--ATGGA
cl2 allele1 GACCTG......-38nt......CTATA
allele2 GACCTG......-38nt......CTATA
G
USP12/46 -dKO
WT ATACCCCCTAAGAAGTTCAT
cl1 allele1 ATACCCCCTATAAGAAGTTC
allele2 ATACCCCCTA-GAAGTTCAT
cl2 allele1 ATACCCCCTAA...+397nt......
allele2 ATACCCCCTA-GAAGTTCAT
WT GACATCCTTCAGGAGGAGAA
cl1 allele1 GACATCCTTCCAGGAGGAGA
allele2 GACATCCTTCCAGGAGGAGA
cl2 allele1 GACATCCTTCCAGGAGGAGA
allele2 GACATCCTTCCAGGAGGAGA

Figure EV2. Related to Fig. 1. Validation of KO clones.

(**A–G**) Agarose gel electrophoresis images show PCR amplification products from the genomic region containing the indicated Cas9 targeting sites of the indicated genes in the parental WT and two independent mouse fibroblast clones (**A–F**) and MDA-MB-231 cell clones (**G**). PCR products were sequenced, analyzed with the Synthego Inference of CRISPR Edits (ICE) analysis tool (Hsiau et al, 2018), and the corresponding alignments are shown on the right panel.

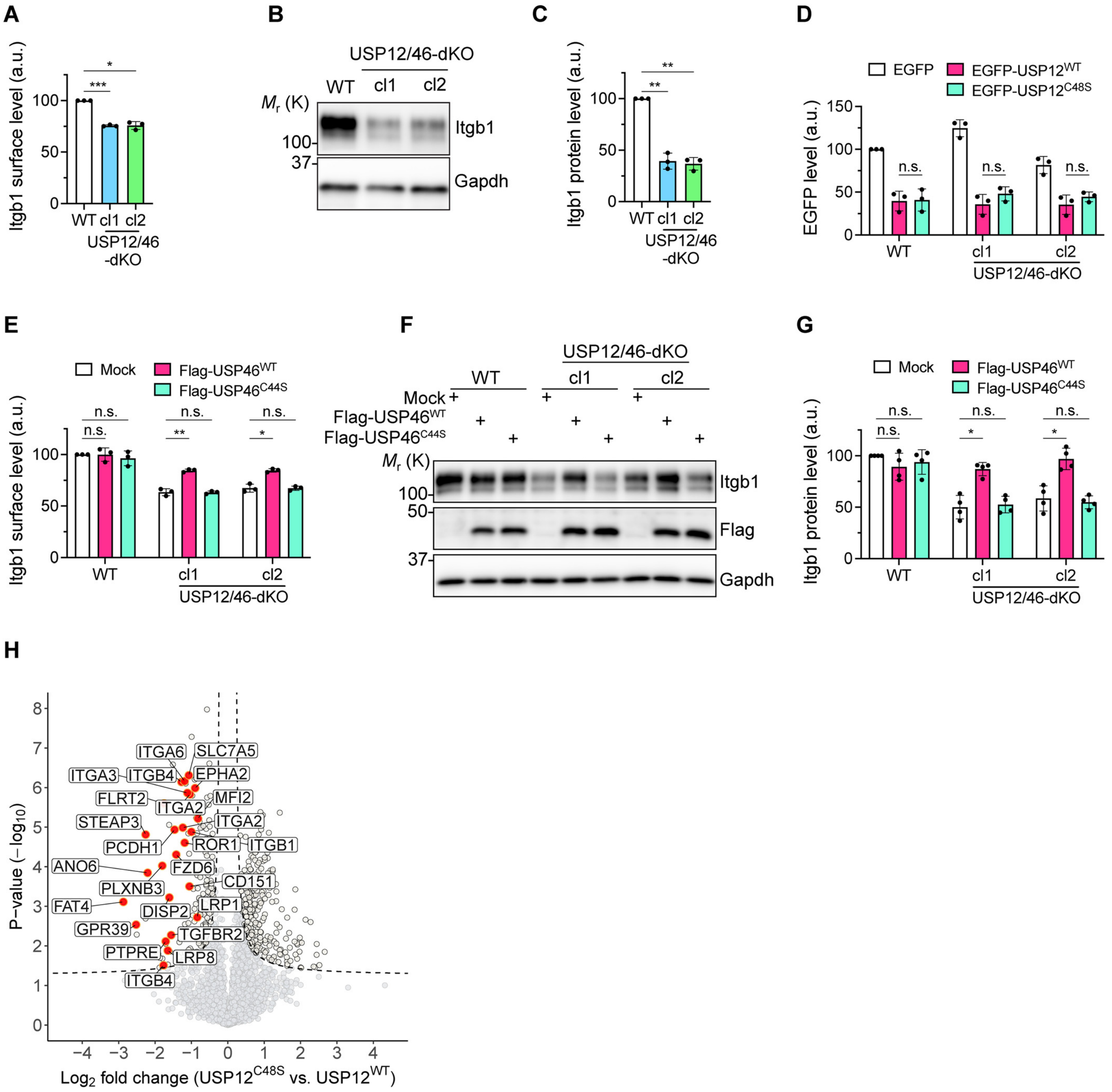
A
Itgb1 surface level (a.u.)
WT cl1 cl2
USP12/46 -dKO
B
USP12/46-dKO
WT cl1 cl2
M_r (K)
100
37
Itgb1
Gapdh
C
Itgb1 protein level (a.u.)
WT cl1 cl2
USP12/46 -dKO
D
EGFP
EGFP-USP12WT
EGFP-USP12C48S
EGFP level (a.u.)
n.s.
WT
cl1
cl2
USP12/46-dKO
E
Mock
Flag-USP46WT
Flag-USP46C44S
Itgb1 surface level (a.u.)
n.s.
WT
cl1
cl2
USP12/46-dKO
F
USP12/46-dKO
WT
cl1
cl2
Mock
Flag-USP46WT
Flag-USP46C44S
M_r (K)
100
50
37
Itgb1
Flag
Gapdh
G
Mock
Flag-USP46WT
Flag-USP46C44S
Itgb1 protein level (a.u.)
n.s.
WT
cl1
cl2
USP12/46-dKO
H
P−value (−log10)
ITGA6
SLC7A5
ITGA3
ITGB4
EPHA2
FLRT2
MFI2
ITGA2
STEAP3
ITGA2
PCDH1
ROR1
ITGB1
ANO6
FZD6
CD151
PLXNB3
FAT4
DISP2
LRP1
GPR39
TGFBR2
PTPRE
LRP8
ITGB4
Log2 fold change (USP12C48S vs. USP12WT)

 © The Author(s)

Figure EV3. Related to Fig. 1. The USP12/46-WDRs complex maintains Itgb1 protein levels.

(**A–C**) Itgb1 surface levels determined by flow cytometry (**A**) and Itgb1 protein levels in cell lysates determined by WB (**B**) with densitometric quantification (**C**) in WT and USP12/46-dKO MDA-MB-231 cells. Gapdh served as a loading control. Statistical analysis was carried out by RM one-way ANOVA with Dunnett's multiple comparison test. In (**A**), statistical significance was tested by comparing WT with USP12/46-dKO cl1 or cl2 MDA-MB-231 cells ($P = 0.0006$ and 0.0126, respectively). In (**C**), statistical significance was tested by comparing WT with USP12/46-dKO cl1 or cl2 MDA-MB-231 cells ($P = 0.0084$ and 0.0048, respectively). $^{*}P < 0.05$; $^{**}P < 0.01$; $^{***}P < 0.001$. Data were shown as Mean ± SD, $n = 3$ independent experiments. (**D**) EGFP fluorescence intensities in WT and USP12/46-dKO fibroblasts stably expressing EGFP, EGFP-USP12^{WT}, or EGFP-USP12^{C48S} determined by flow cytometry. Statistical analysis was carried out by ordinary two-way ANOVA with Šidák's multiple comparison test comparing the EGFP-USP12^{WT} group and EGFP-USP12^{C48S} group in WT fibroblasts ($P = 0.9987$); in USP12/46-dKO cl1 fibroblasts ($P = 0.4423$) and in USP12/46-dKO cl2 fibroblasts ($P = 0.6692$). n.s. not significance. Data were shown as Mean ± SD, $n = 3$ independent experiments. (**E–G**) Itgb1 surface levels determined by flow cytometry (**E**) and Itgb1 protein levels in cell lysates determined by WB (**F**) with densitometric quantification (**G**) in WT and USP12/46-dKO fibroblasts stably expressing FLAG-USP46^{WT} or FLAG-USP46^{C44S}. Mock-transduced cells (Mock) served as control. Gapdh served as a loading control. Statistical analysis was carried out by RM two-way ANOVA with Dunnett's multiple comparison test. In (**E**), statistical significance was tested comparing the Mock group with Flag-USP46^{WT} or Flag-USP46^{C44S} group in WT fibroblasts ($P = 0.9990$ and 0.6491, respectively); in USP12/46-dKO cl1 fibroblasts ($P = 0.0045$ and 0.9669, respectively); and in USP12/46-dKO cl2 fibroblasts ($P = 0.0329$ and 0.9996, respectively). In (**G**), statistical significance was tested comparing the Mock group with Flag-USP46^{WT} or Flag-USP46^{C44S} group in WT fibroblasts ($P = 0.3116$ and 0.5586, respectively); in USP12/46-dKO cl1 fibroblasts ($P = 0.0127$ and 0.4378, respectively); and in USP12/46-dKO cl2 fibroblasts ($P = 0.0168$ and 0.7514, respectively). $^{*}P < 0.05$; $^{**}P < 0.01$; n.s. not significant. Data were shown as Mean ± SD. (**E**) $n = 3$; (**F**, **G**) $n = 4$ independent experiments. (**H**) Volcano plot of the cell surface proteome of USP12/46-dKO MDA-MB-231 cells expressing EGFP-USP12^{C48S} versus EGFP-USP12^{WT} identified by label-free MS. *P* values are determined using two-sided permuted *t*-test with 250 randomizations. The black dashed line indicates the significance cutoff (FDR:0.05, S0:0.1) estimated by the Perseus software. $n = 4$ biological replicates. Arbitrarily selected cell surface receptors are highlighted in red.

© The Author(s)

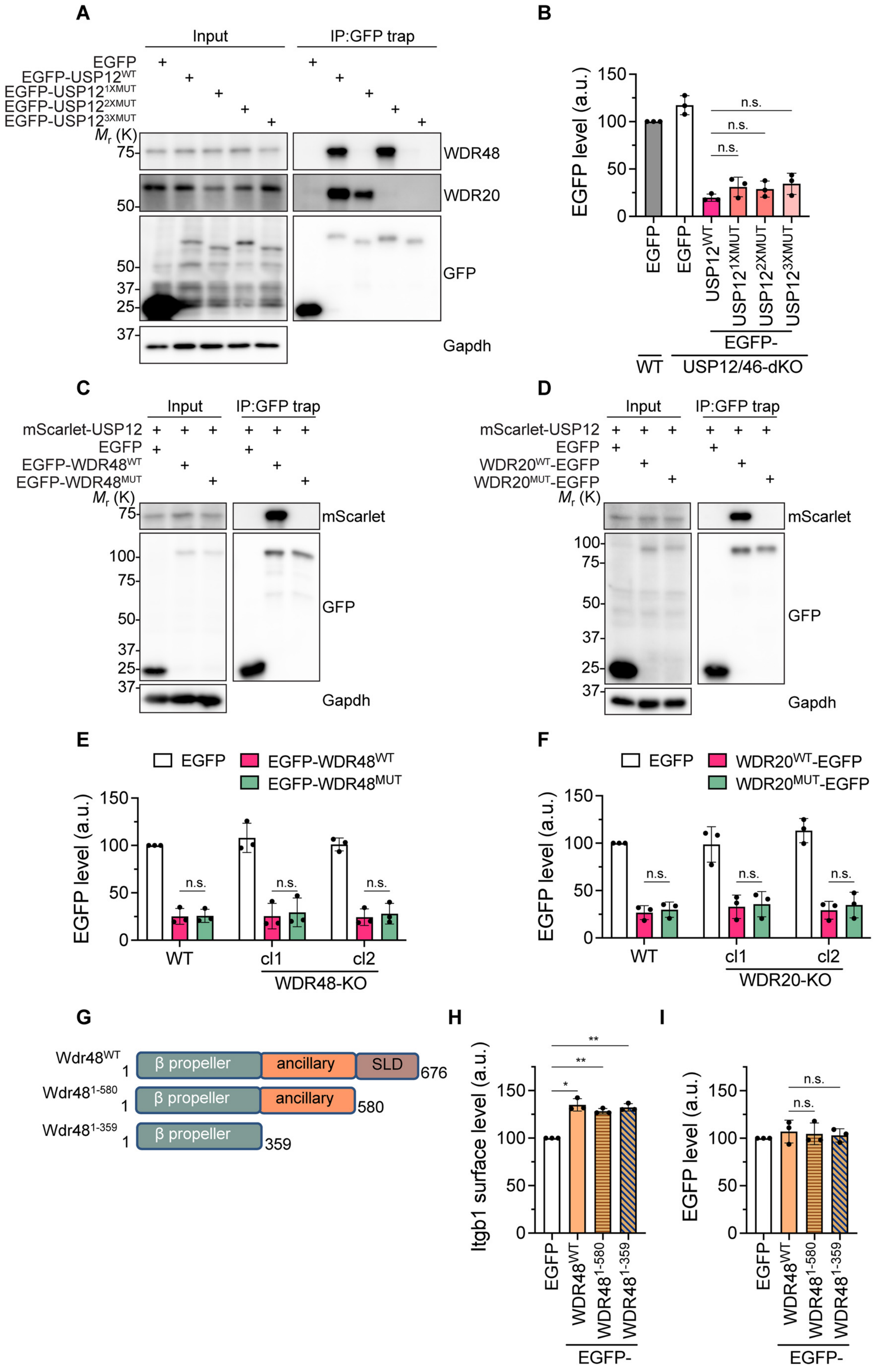
A
Input
IP:GFP trap
EGFP
EGFP-USP12WT
EGFP-USP121XMUT
EGFP-USP122XMUT
EGFP-USP123XMUT
Mr (K)
WDR48
WDR20
GFP
Gapdh
B
EGFP level (a.u.)
n.s.
EGFP
USP12WT
USP121XMUT
USP122XMUT
USP123XMUT
EGFP-
WT
USP12/46-dKO
C
Input
IP:GFP trap
mScarlet-USP12
EGFP
EGFP-WDR48WT
EGFP-WDR48MUT
mScarlet
GFP
Gapdh
D
WDR20WT-EGFP
WDR20MUT-EGFP
E
EGFP-WDR48WT
EGFP-WDR48MUT
WT
cl1
cl2
WDR48-KO
F
WDR20-KO
G
Wdr48WT
Wdr481-580
Wdr481-359
β propeller
ancillary
SLD
676
580
359
H
Itgb1 surface level (a.u.)
WDR48WT
WDR481-580
WDR481-359
I

Figure EV4. Related to Fig. 2. Characterization of binding-deficient USP12, WDR48, and WDR20 mutants.

(**A**) GFP immunoprecipitation (GFP IP) from USP12/46-dKO fibroblasts transiently expressing EGFP, EGFP-USP12^{WT}, EGFP-USP12^{1XMUT}, EGFP-USP12^{2XMUT}, or EGFP-USP12^{3XMUT} analyzed by WB for indicated proteins. Gapdh served as a loading control. Representative images from three independent experiments are shown. (**B**) EGFP fluorescence in WT and USP12/46-dKO fibroblasts transiently expressing EGFP, EGFP-USP12^{WT}, EGFP-USP12^{1XMUT}, EGFP-USP12^{2XMUT}, or EGFP-USP12^{3XMUT} determined by flow cytometry. Statistical analysis was carried out by ordinary one-way ANOVA with Dunnett's multiple comparison test comparing the EGFP-USP12^{WT} group with EGFP-USP12^{1XMUT}, EGFP-USP12^{2XMUT}, or EGFP-USP12^{3XMUT} group in USP12/46-dKO cl1 fibroblasts ($P = 0.3384$, 0.4963, and 0.1783, respectively). n.s. not significant. Data were shown as Mean ± SD, $n = 3$ independent experiments. (**C**) GFP IP from USP12/46-dKO fibroblasts stably expressing mScarlet-USP12 and transiently expressing EGFP, EGFP-WDR48^{WT}, or EGFP-WDR48^{MUT} analyzed by WB for indicated proteins. Gapdh served as a loading control. Representative images from three independent experiments are shown. (**D**) GFP IP from USP12/46-dKO fibroblasts stably expressing mScarlet-USP12 and transiently expressing EGFP, WDR20^{WT}-EGFP, or WDR20^{MUT}-EGFP analyzed by WB for indicated proteins. Gapdh served as a loading control. Representative images from three independent experiments are shown. (**E**) EGFP fluorescence in WT and WDR48-KO fibroblasts transiently expressing EGFP, EGFP-WDR48^{WT}, or EGFP-WDR48^{MUT} determined by flow cytometry. Statistical analysis was carried out by ordinary two-way ANOVA with Šidák's multiple comparison test comparing the EGFP-WDR48^{WT} group with EGFP-WDR48^{MUT} group in WT fibroblasts ($P = 0.9999$); in WDR48-KO cl1 fibroblasts ($P = 0.9630$); and in WDR48-KO cl2 fibroblasts ($P = 0.9701$). n.s. not significant. Data were shown as Mean ± SD, $n = 3$ independent experiments. (**F**) EGFP fluorescence in WT and WDR20-KO fibroblasts transiently expressing EGFP, WDR20^{WT}-EGFP, or WDR20^{MUT}-EGFP determined by flow cytometry. Statistical analysis was carried out by ordinary two-way ANOVA with Šidák's multiple comparison test comparing the WDR20^{WT}-EGFP group with the WDR20^{MUT}-EGFP group in WT fibroblasts ($P = 0.9814$); in WDR20-KO cl1 fibroblasts ($P = 0.9870$); and in WDR20-KO cl2 fibroblasts ($P = 0.9070$). n.s. not significant. Data were shown as Mean ± SD, $n = 3$ independent experiments. (**G**) Domain organization of the WT WDR48 and WDR48 domain-deletion mutants. (**H**, **I**) Itgb1 surface levels (**H**) and EGFP fluorescence (**I**) in WDR48-KO fibroblasts stably expressing EGFP, EGFP-WDR48^{WT}, EGFP-WDR48$^{1\text{-}580}$, or EGFP-WDR48$^{1\text{-}359}$ determined by flow cytometry. Statistical analysis was carried out by RM one-way ANOVA with Dunnett's multiple comparison test. In (**H**), statistical significance was tested comparing the EGFP group with EGFP-WDR48^{WT}, EGFP-WDR48$^{1\text{-}580}$, or EGFP-WDR48$^{1\text{-}359}$ group ($P = 0.0216$, 0.0065, and 0.0093, respectively). In (**I**), statistical significance was tested by comparing the EGFP-WDR48^{WT} group with EGFP-WDR48$^{1\text{-}580}$ or EGFP-WDR48$^{1\text{-}359}$ group ($P = 0.8951$ and 0.7411, respectively). $^*P < 0.05$; $^{**}P < 0.01$; n.s. not significant. Data were shown as Mean ± SD, $n = 3$ independent experiments.

© The Author(s)

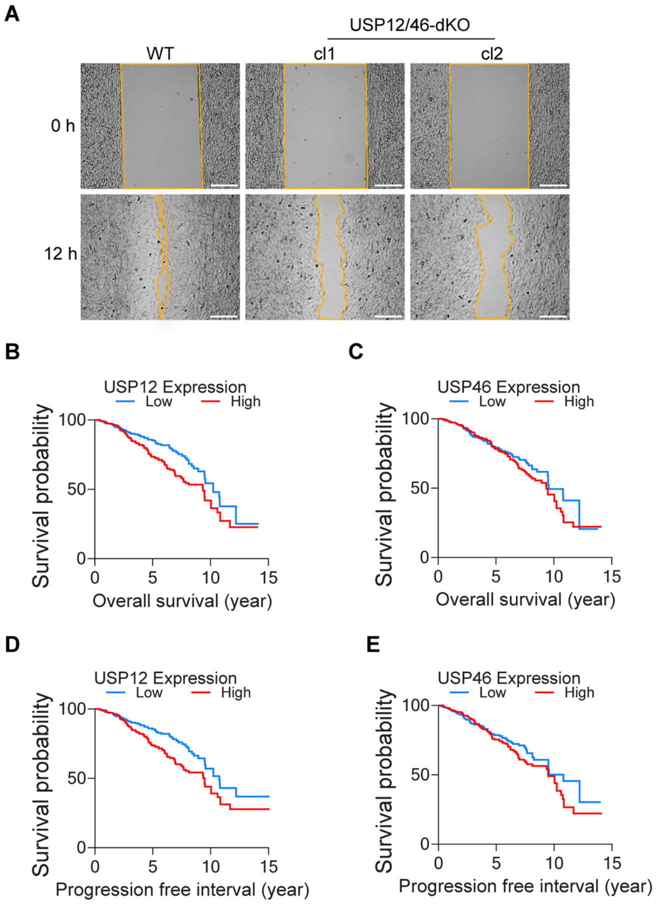

Figure EV5. Related to Fig. 8. USP12 and USP46 are not favorable for prognosis in cancer patients.

(**A**) Representative images of the in vitro wound healing assay showing WT and USP12/46-dKO fibroblasts migrating on FN-coated 2D surfaces at 0 and 12 h. Lines mark the leading edge of cell migration towards the wound. Scale bar, 200 μm. (**B–E**) Kaplan–Meier plot of the overall survival (**B**, **D**) and progression-free interval (**C**, **E**) of breast cancer patients with high (red line) or low (blue line) gene expression *USP12* (**B**, **C**) or *USP46* (**D**, **E**) levels. The GDC TCGA dataset obtained from the UCSC Xena project (Goldman et al, 2020) was used. Two-group risk model with a cut-off at the median was applied. *P* values were calculated by log-rank test. The *P* values in (**B–E**) are 0.0059, 0.3898, 0.0058, and 0.3927, respectively.

 © The Author(s)
